# Biocatalytic ulvan degradation by *Pseudoalteromonas marina*: exploring a marine polysaccharide bioconversion system

**DOI:** 10.1186/s12934-026-02969-0

**Published:** 2026-02-27

**Authors:** Navindu Dinara Gajanayaka, Eunyoung Jo, Minthari Sakethanika Bandara, Yeon-Ju Lee, Jaewon Lee, Svini Dileepa Marasinghe, Jonathan Sathyadith, Tae-Yang Eom, Gun-Hoo Park, Chulhong Oh, Youngdeuk Lee

**Affiliations:** 1 Jeju Bio Research Center, Korea Institute of Ocean Science and Technology (KIOST), 2670 Ilju-dong-ro, Gujwa-eup, Jeju-si, 63349 Republic of Korea; 2 Department of Marine Technology & Convergence Engineering (Marine Biotechnology), KIOST School, University of Science and Technology (UST), Daejeon, Republic of Korea; 3 Marine Natural Products Chemistry Laboratory, Korea Institute of Ocean Science and Technology (KIOST), Busan, 49111 Republic of Korea

**Keywords:** Ulvan depolymerization, Ulvan utilization pathway, Ulvan lyases, *Pseudoalteromonasmarina*, Recombinant protein expression

## Abstract

Ulvan depolymerization by marine bacteria is a specialized metabolic capability with significant implications for marine carbon cycling and biotechnological applications. While ulvan degradation systems have been extensively characterized in Bacteroidota, their occurrence and mechanisms in Pseudomonadota remain poorly understood. This study proposed a putative ulvan utilization system in the marine bacterium *Pseudoalteromonas marina* PUA1001, representing a notable metabolic expansion beyond the typical Pseudomonadota repertoire. Comparative genomic analysis with the closely related strain *P. marina* mano4 (97.5% OrthoANI) revealed a complete absence of the ulvan utilization locus in mano4, suggesting a possible role and strain-level metabolic specialization. The model PUA1001 ulvan utilization locus comprises three polysaccharide lyases (PL24, PL25, PL40), seven glycoside hydrolases, including previously unreported GH106 and GH115 families, two sulfatases, and seven accessory genes, predicted to enable stepwise depolymerization of ulvan to monosaccharides. Biochemical characterization of the three recombinantly expressed ulvan lyases revealed optimal activities at 35–50 °C and pH 8.0, with significant Mn²⁺-dependent activity enhancement (1.25 − 2 fold). Molecular docking analysis of PmUL24 and PmUL25 confirmed conservation of the His/Tyr-dependent β-elimination mechanism. This polysaccharide utilization system may contribute to the ecological success of *Pseudoalteromonas* species in ulvan-rich marine environments, highlighting adaptive flexibility and strain-specific potential for exploiting distinct polysaccharide niches within marine ecosystems.

**Supplementary Information:**

The online version contains supplementary material available at 10.1186/s12934-026-02969-0.

## Introduction

Marine environments create severe survival challenges for organisms spanning from microscopic to macroscopic taxa due to environmental extremes, intense intraspecific and interspecific competition, predation, and growing human impacts [1–3]. In response to these harsh conditions, marine organisms have developed highly specialized and adaptive metabolic pathways over millions of years of evolutionary refinement. Marine bacteria have excelled in this evolutionary arms race, evolving to inhabit virtually every oceanic niche with remarkable strategies to thrive in nutrient-scarce ocean waters [4].

Marine environments are rich in complex polysaccharides, especially in algal cell walls, while readily available simple sugars are often scarce [5]. This nutrient imbalance has driven the evolution of highly substrate-specific and specialized enzymatic cascade systems, along with supportive genes in microorganisms, to recognize specific polymers, depolymerize structurally intricate polymers, transport the degraded products across membranes, and convert them into usable energy and carbon. Additionally, microorganisms are the key protagonists in nutrient cycling, especially in coastal waters, where they transform algal biomass into forms that can be utilized by higher trophic levels [6]. These sophisticated biochemical adaptations of marine microbes enable marine life to not only survive but thrive in one of Earth’s most demanding ecosystems.

Polysaccharide depolymerization to utilizable simple sugars is an intricate process that primarily depends on specific genomic regions known as polysaccharide utilization loci (PULs) [7]. PULs were first identified in *Bacteroidetes thetaiotaomicron* VPI-5482, a human gut-associated bacterium [8]. Generally, the phylum Bacteroidetes is well known for complex carbohydrate degradation and often inhabits symbiotic relationships with algae, plants, and animal gastrointestinal tracts [9]. The diverse array of carbohydrate-active enzymes (CAZymes) underlies successful polymer breakdown. These gene clusters typically encode surface-related carbohydrate-binding proteins that identify target polysaccharides, TonB-dependent transporters that import depolymerized oligosaccharides across the outer membrane, and CAZymes that depolymerize the polymer [10]. In marine bacteria, PULs are often highly substrate specific, with genes that are well adapted according to the habitat environment of the species [11]. Compared with other PULs, ulvan-dependent PULs are the least documented. In 2019, ulvan-dependent utilization loci were identified based on the marine bacterium *Formosa agariphila* KMM 3901 [12]. Ulvan lyases are enzymes that can depolymerize ulvan into oligosaccharides. To date, the CAZy database has recorded five different ulvan lyase families: polysaccharide lyase family 24, 25, 28, 37, & 40 (PL24, PL25, PL28, PL37, and PL40, respectively) (http://www.cazy.org/). Ulvan lyases cleave β-(1→4) glycosidic linkages via a β-elimination reaction, resulting in the formation of an unsaturated sugar ring at one end and a reducing sugar end at the other [13].

Among marine polysaccharides, ulvan has recently attracted significant attention from both the microbiome and scientific communities due to the intensive growth of available resources. From a biological perspective, microbes have evolved to efficiently utilize ulvan’s abundant nutrients; from an industrial standpoint, the scientific community recognizes its potential for various commercial applications. Ulvan is a sulfated, water-soluble, anionic, mostly linear polysaccharide found in the cell walls of algae in the order Ulvales [14]. *Ulva* spp., commonly known as sea lettuce, is one of the most prominent species that thrives in intertidal and subtidal coastal zones and has recently been forming vast algal blooms in eutrophic waters. Thereby, these algal blooms generate substantial amounts of biomass, constituting a largely underexploited renewable biological resource with significant potential for sustainable biotechnological applications. The structural backbone of ulvan consists of repeating disaccharide units containing L-rhamnose-3-sulfate (Rha3S) linked to uronic acids (D-glucuronic acid (GlcA) and L-iduronic acid (IdoA)), or D-xylose (Xyl), resulting in diverse structural configurations and distinct biological characteristics. The main structural motifs are ulvanobiuronic acid type A [→4)-β-D-GlcA-(1→4)-α-L-Rha3S-(1→] and type B [→4)-α-L-IdoA-(1→4)-α-L-Rha3S-(1→] [15]. The unique physicochemical properties of ulvan result from variations in sulfation degree and position, combined with the specific ratio of uronic acids and xylose in the linear backbone. However, the structural configuration of ulvan is not static; it can vary with environmental conditions. Factors such as water temperature, salinity, light availability, and nutrient levels can be directly dependent on the monosaccharide composition, sulfation amount, and the molecular weight of the polymer [16, 17]. Furthermore, this structural composition directly impacts microbial diversity and the degradability of ulvan by microorganisms, since bacteria inhabiting ulvan-associated environments must deploy an array of enzymes that precisely target the specific linkage types and structural patterns within ulvan structure. Therefore, understanding the biochemical and ecological aspects of ulvan diversity and degradation is crucial both for advancing our knowledge of microbial metabolism and for optimizing diverse biotechnological applications.

Ulvan-utilizing microbes can produce oligosaccharides and monosaccharides that are released into the surrounding environment, where they can be rapidly assimilated by microorganisms or sequestered through biological carbon pumps. This natural catabolic process has sparked growing interest in harnessing ulvan lyases to produce specific ulvan oligosaccharide fractions with distinct bioactivities. However, while ulvan with different bioactivities, including antioxidant, anticoagulant, antiviral, and immunomodulatory effects, is widely applied in industry, there remains a significant knowledge gap regarding the application of ulvan oligosaccharides with specific bioactivities [17]. Comparatively, a major disadvantage of ulvan polysaccharides is their high viscosity, which hinders their applications compared to ulvan oligosaccharides [18].

Marine bacteria represent a valuable reservoir of CAZymes with significant potential as biocatalysts for industrial applications. The discovery and characterization of these enzymes could enable the development of distinct and controlled depolymerization of complex polymers. Despite recent progress, significant knowledge gaps remain regarding the diversity and distribution of ulvan-degrading microbes, ulvan metabolism, and their ecological roles in coastal waters. Therefore, we employed a bioinformatics approach to understand ulvan utilization by *Pseudoalteromonas* sp., including comparative genomic analysis, draft genome analysis, gene domain structure analysis, and subcellular localization prediction. Additionally, we provide gene characterization of three different novel ulvan lyases representing three distinct polysaccharide lyase families.

## Materials and methods

### Screening and isolation of ulvan-degrading marine bacteria

In our previous study, we collected partially decomposed *Ulva*-associated water samples from Jeju Island, South Korea, a frequent green tide occurring area in Eastern Asia [19]. Following the process described in that study, we successfully identified and screened ulvan-hydrolyzing microbes using 10% ulvan plates. Following multiple confirmation steps to verify ulvan hydrolyzing activity, we isolated single colonies and amplified their 16 S rRNA genes for species identification (Macrogen, Seoul, South Korea).

### Draft genome analysis, identification, and phylogenetic analysis of ulvan lyases

The draft genome of the isolated ulvan-hydrolyzing strain was sequenced and analyzed by CJ Bioscience, Inc. (Seoul, South Korea). Putative ulvan lyases were identified through comparative analysis using the Basic Local Alignment Search Tool (BLAST Protein BLAST: search protein databases using a protein query) and the CAZy database (CAZy - PL). All identified ulvan lyase sequences were deposited in the National Center for Biotechnology Information (NCBI) GenBank database and assigned individual accession numbers. Signal peptide prediction and transmembrane helix topology analysis were performed using SignalP 6.0 [20] and DeepTMHMM version 1.0.24 [21], respectively. Protein domain architecture was predicted using the Simple Modular Architecture Research Tool version 9 (SMART SMART: Main page) [22] and InterPro version 101.0 databases InterPro [23]. All characterized ulvan lyases currently available in the CAZy database were retrieved for phylogenetic analysis. Multiple sequence alignment was performed using the ClustalW algorithm implemented in Molecular Evolutionary Genetics Analysis (MEGA X software version 12). A phylogenetic tree was constructed based on the aligned sequences using the maximum likelihood method to infer evolutionary relationships among ulvan lyases [24]. The phylogenetic tree was visualized and annotated using the Interactive Tree Of Life (iTOL) version 6 web based platform [25].

### Cloning, overexpression, and purification of recombinant proteins

Genomic DNA extracted from the ulvan-degrading marine bacterial strain (PUA1001) was used as the template for polymerase chain reaction (PCR) amplifications. Genomic DNA was extracted using the AccuPrep^®^ Genomic DNA Extraction Kit (Bioneer, Daejeon, South Korea) according to the manufacturer’s instructions. Three ulvan lyase genes, each representing distinct polysaccharide lyase families, were selected as targets for PCR amplification. All the PCRs were conducted without the signal peptides or transmembrane domains from the original sequences. Gene-specific primers were designed for PCR amplification of *pmul24*, *pmul25*, and *pmul40*. The primer sequences were as follows: *pmul24* forward 5’-ATCGAAGGTCGTCATATGCACGCAGTGGTTGAATTAGAGCAAC-3’ and reverse 5’-TTGTTAGCAGCCGGATCCTTAGTTTTCAAACAATACATTAAACTCTGATATATCAGGCCAG-3’; *pmul25* forward 5’-ATCGAAGGTCGTCATATGGGTTGTAGTTATAATCAACCAAAGAATGATAAC-3’ and reverse 5’-TTGTTAGCAGCCGGATCCTTAATTGCCAATTTCTGACTTTAAGCGGGC-3’; *pmul40* forward 5’-ATCGAAGGTCGTCATATGACAGAGGTAGAGCGCCCTTC-3’ and reverse 5’-TTGTTAGCAGCCGGATCCTTACATTTTCATGTAGGCTGAATTTTTATAAAAGGTTG-3’. PCR products were purified, digested with *NdeI* and *BamHI* restriction endonucleases, and ligated into the corresponding sites of the linearized pET-16b expression vector (NovaGen, Madison, WI, USA). Recombinant plasmids were transformed into *Escherichia coli* DH5α competent cells using the heat shock method [26]. Transformants were selected on Luria−Bertani (LB) agar plates supplemented with ampicillin (amp) (100 µg/mL) and incubated overnight at 37 °C. An individual colony was picked and inoculated into 4 mL of LB/amp overnight at 37 °C with constant shaking at 180 rpm. Positive transformants were validated by extracting the plasmid DNA (Bioneer, Daejeon, South Korea) and performing Sanger sequencing (Macrogen, Seoul, South Korea) to verify correct insert orientation and sequence. Sequence-verified plasmids were transformed into *E. coli* BL21(DE3) competent cells to enable recombinant protein expression. Protein expression was induced at an optical density of 0.6 − 0.8 (OD_600_) by adding isopropyl-β-d-thiogalactopyranoside to a final concentration of 0.1 mM. Induced cells were incubated at 20 °C with shaking at 180 rpm in LB/amp media. Following an 18-h induction period, cells were collected by centrifugation at 8,000 × g for 5 min at 4 °C. The cell pellets were resuspended in binding buffer from the Novagen His-Tag Purification Kit and frozen at -20 °C overnight. The following day, cell pellets were thawed and lysed by sonication on ice at 85% amplitude. The soluble fraction was separated from cell debris by centrifugation at 13,000 × g for 30 min at 4 °C. The N-terminal His-tagged protein was purified from the clarified lysate using a Novagen His-Tag Purification Kit (Novagen, Madison, WI, USA). Protein yield was quantified with a bicinchoninic acid assay kit (Thermo Fisher Scientific, Waltham, MA, USA), and sample purity was confirmed using 12% (w/v) sodium dodecyl sulfate polyacrylamide gel electrophoresis (SDS-PAGE).

### Biochemical characterization of ulvan lyases

To evaluate thermal stability, recombinant PmUL24, PmUL25, and PmUL40 were preincubated at three temperatures (25 °C, 35 °C, and 45 °C) for various time intervals (2, 4, 6, 8, and 10 min), followed by residual activity measurements. Temperature optima were determined by assaying enzyme activity across a range of 25–60 °C. pH optima were identified using overlapping buffer systems at a final concentration of 50 mM: citrate-phosphate buffer (pH 3 − 7), phosphate buffer (pH 6 − 8), Tris-HCl (pH 7 − 10), and glycine-NaOH (pH 9 − 10). The effect of ionic strength on enzyme activity was examined using NaCl concentrations from 0 to 1,000 mM. To assess metal ion effects on enzyme activity, various divalent cations (Cu^2+^, K^+^, Zn^2+^, Mg^2+^, Ca^2+^, Fe^2+^, and Mn^2+^) were added to the reaction mixture at final concentrations of 2.5, 5, and 10 mM, and activity was measured after 5 min of incubation. All enzyme activities were measured using a modified 3,5-dinitrosalicylic acid assay following the established protocol [27, 28]. Data represent mean values ± standard deviation from three independent experiments. For characterization studies, ulvan polysaccharide was extracted from *Ulva* sp. and used to assess each ulvan lyase [19].

Purified recombinant enzymes PmUL24, PmUL25, and PmUL40 were individually incubated with commercial ulvan [1% (w/v)] (YU11689 BIOSYNTH BiosynthLtd., United Kingdom) at room temperature for 24 h. Enzymatic reactions were terminated by heat inactivation, and the products were prepared for liquid chromatography-mass spectrometry (LC-MS) analysis.

Hydrolyzed samples were filtered (0.45 μm pore size) and diluted to 100 ppm with MS-grade water. Chromatographic separation was achieved using an Acquit UPLC BEH Amide column (2.1 × 100 mm, 1.7 μm particle size) with gradient elution using 10 mM ammonium acetate in water/acetonitrile (95:5, v/v) at pH 6.82 as Eluent A and 10 mM ammonium acetate in acetonitrile/water (95:5, v/v) at pH 8.22 as Eluent B. The gradient program was: 0.00 min (2% A, 98% B), 6.00 min (40% A, 60% B), 7.60 min (40% A, 60% B), 7.70 min (2% A, 98% B), and 15.0 min (2% A, 98% B). The flow rate was 0.25 mL/min, the column temperature was maintained at 60 °C, and the sample injection volume was 5.0 µL. Mass spectrometric analysis was performed on a UHPLC (SCIEX ExionLC AD machine, MA, USA) coupled with an X500R Q-TOF system (SCIEX, Framingham, MA, USA) operated in negative electrospray ionization (ESI−) mode with the following parameters: ion mode ESI−, ion source gas 1 at 50 psi, ion source gas 1 at 55 psi, curtain gas at 25 psi, temperature at 550 °C, spray voltage at − 4500 V, declustering potential at − 80 V, and collision energy at − 10 V. Depolymerization products were characterized based on their mass-to-charge ratios (m/z) and MS/MS fragmentation patterns with accumulation time of 0.025 s, declustering potential of − 80 V, and collision energy of − 35 V.

### Ulvan lyases structural analysis

Homology modeling of ulvan lyases PmUL24 and PmUL25 was conducted using the SWISS-MODEL web tool (https://swissmodel.expasy.org/). The structural models were predicted using templates from the Protein Data Bank (PDB; RCSB PDB: Homepage). PmUL40 could not be modeled due to the lack of suitable templates with available X-ray crystallography or cryo-EM structures. The structure of PmUL24 was predicted using PDB: 6BYP as a template, while PmUL25 was modeled using PDB: 5UAM [29, 30].

Additionally, the predicted structures were docked with an ulvan oligomer DP4 ligand (ΔUA-R3S-GlcA-R3S) to determine the ulvan lyases and ligand interactions. The ligand conformer was generated using the Neurosnap Conformer Generator web server ( Use Conformer Generator Online | Neurosnap). Molecular docking was performed using AutoDock Tools 1.5.6 and AutoDock Vina 1.1.2 (Trott and Olson, 2010) to determine the interactions between the DP4 ligand and the receptors (PmUL24 and PmUL25). The binding pocket of each ulvan lyase was identified through sequence alignment with template ulvan lyases. A grid box with dimensions of 40 × 40 × 40 Å was generated around the active site using a grid spacing of 0.375 Å to define the docking region. The center coordinates for the grid box were set as follows: PmUL24 (x = 112.657, y = 50.162, z = -11.897) and PmUL25 (x = 12.232, y = 89.998, z = 26.321). To obtain accurate docking results, the energy range was set to 4 kcal/mol, and the exhaustiveness parameter was set to 50. The best docking pose, representing the ligand-receptor interaction with the lowest binding energy, was visualized in 2D and 3D using Discovery Studio 2024 (BIOVIA, San Diego, CA, USA).

### Comparative genome analysis and prediction of ulvan utilization loci

The draft genome sequence of *Pseudoalteromonas marina* PUA1001 was aligned against genomes of other *Pseudoalteromonas* spp. All genomes and genome comparative tools were obtained from the EZBioCloud database (EzBioCloud.net | Search about Bacteria or Archaea). According to EZBioCloud, the taxon names, project accession numbers, strain names, and sources of bacteria isolation are as follows (Table 1).

**Table 1:** Bacterial strains used for comparative genomic analysis

Taxon name	Project accession (According to EZBioCloud)	Strain name	Source of isolation
*Pseudoalteromonas* * marina*	31,464.PUA1001.1	PUA1001	Ulva-associated water (This study)
*Pseudoalteromonas* * marina*	GCA_000238335.3	mano4	Tidal flat sediment
*Pseudoalteromonas* * arctica*	GCA_000238395.4	A 37-1-2	Arctic seawater
*Pseudoalteromonas* *carrageenovor*a	GCA_900239935.1	ATCC 43,555	-
*Pseudoalteromonas* *distincta*	GCA_000814675.1	ATCC 700,518	Marine sponge

The table lists strain identifiers, taxonomic classifications, genome project accession numbers, and isolation sources retrieved from the EzBioCloud database. These strains were selected for comparative analysis of ulvan lyase gene distribution and polysaccharide degradation capabilities across *Pseudoalteromonas* species

Pairwise genome relatedness was determined using the Orthologous Average Nucleotide Identity (OrthoANI) algorithm, which calculates ANI values based on orthologous gene segments between genomes [31]. The reciprocal best hit algorithm was employed for ortholog detection across all genomes. The resulting pairwise orthologous relationships were compiled into an ortholog matrix (POM) using PUA1001 as the reference genome [32, 33]. Following ortholog detection, all protein-coding sequences (CDSs) were clustered into Pan genome Orthologous Groups to define non-redundant gene families. The pan genome was stratified into three categories: (i) core genes present in all genomes, typically encoding essential or housekeeping functions; (ii) accessory genes with variable distribution across genomes; and (iii) singleton genes unique to individual strains. Gene frequency distribution across the dataset was visualized using a gene frequency plot [34, 35]. The ortholog matrix data were used to generate a Venn diagram illustrating the pan genome structure, including core genes (present in all genomes), accessory genes (present in some genomes), and unique genes (strain-specific) [36].

Contig 3 of strain PUA1001 was selected for detailed characterization based on pan genome and ortholog matrix analyses, which revealed significant enrichment of singleton genes within this region. Each gene encoded on contig 3 was systematically analyzed for protein localization signals and structural features. Signal peptide prediction was performed to identify classical signal peptides (SignalP-6.0 SignalP 6.0 - DTU Health Tech - Bioinformatic Services) and lipoprotein signal peptides (LipoP-1.0 LipoP 1.0 - DTU Health Tech - Bioinformatic Services). Transmembrane topology was assessed for both α-helical transmembrane domains and β-barrel outer membrane proteins (DeepTMHMM-1.0 DeepTMHMM 1.0 - DTU Health Tech - Bioinformatic Services, TMHMM-2.0 TMHMM 2.0 - DTU Health Tech - Bioinformatic Services). Conserved functional domains were identified using SMART and InterProScan databases.

Subcellular localization predictions were performed using multiple computational tools to ensure robust predictions: PRED-TMBB for β-barrel outer membrane proteins (PRED-TMBB: Prediction of TransMembrane Beta-Barrel Proteins), DeepLoc-2.0 (DeepLoc 2.0 - DTU Health Tech - Bioinformatic Services), PSORTb (PSORTb Subcellular Localization Prediction Tool - version 3.0), BUSCA (BUSCA - Bologna Biocomputing Group), PSLpred (Pslpred: A svm based method for the subcellular localization of prokaryotic proteins), and Gneg-mPLoc (Gneg-mPLoc server) for general subcellular localization of each protein. Final subcellular localization assignments for each gene were determined by consensus analysis across all prediction tools. Putative gene functions were assigned based on NCBI BLAST homology searches against reference databases. Integration of structural features, localization predictions, and functional annotations enabled the identification and characterization of a complete putative ulvan utilization loci. Based on these analyses, a comprehensive model of the ulvan degradation pathway was proposed and illustrated.

## Results

### Screening and isolation of ulvan-degrading marine bacteria

To identify marine bacteria capable of utilizing ulvan as a sole carbon source, enrichment cultures were established on seawater containing 10% (w/v) ulvan. Following incubation at 25 °C for 2–3 days, distinct zones of clearing were observed around bacterial colonies, indicating ulvan degradation activity (Supplementary Fig. 1). Bacteria exhibiting clearing zones were isolated through serial streaking and subsequently identified by 16 S rRNA gene sequencing. The isolate designated PUA1001 exhibited 99.6% sequence similarity to *Pseudoalteromonas marina*, confirming its taxonomic assignment to this species.

### Draft genome analysis, identification, and phylogenetic analysis of ulvan lyases

The draft genome assembly of *P. marina* PUA1001 comprised three contigs spanning 4,230,469 bp with an average GC content of 39.6%. Automated annotation predicted 3,711 CDSs across the genome. Three ulvan lyase genes were identified on contig 3, belonging to polysaccharide lyase families 24 (PL24), 25 (PL25), and 40 (PL40). The genes encode open reading frames of 3,168 bp (*pmul24*), 1,413 bp (*pmul25*), and 2,565 bp (*pmul40*), corresponding to predicted proteins of 1,056, 471, and 855 amino acids, respectively.

**Fig. 1:**
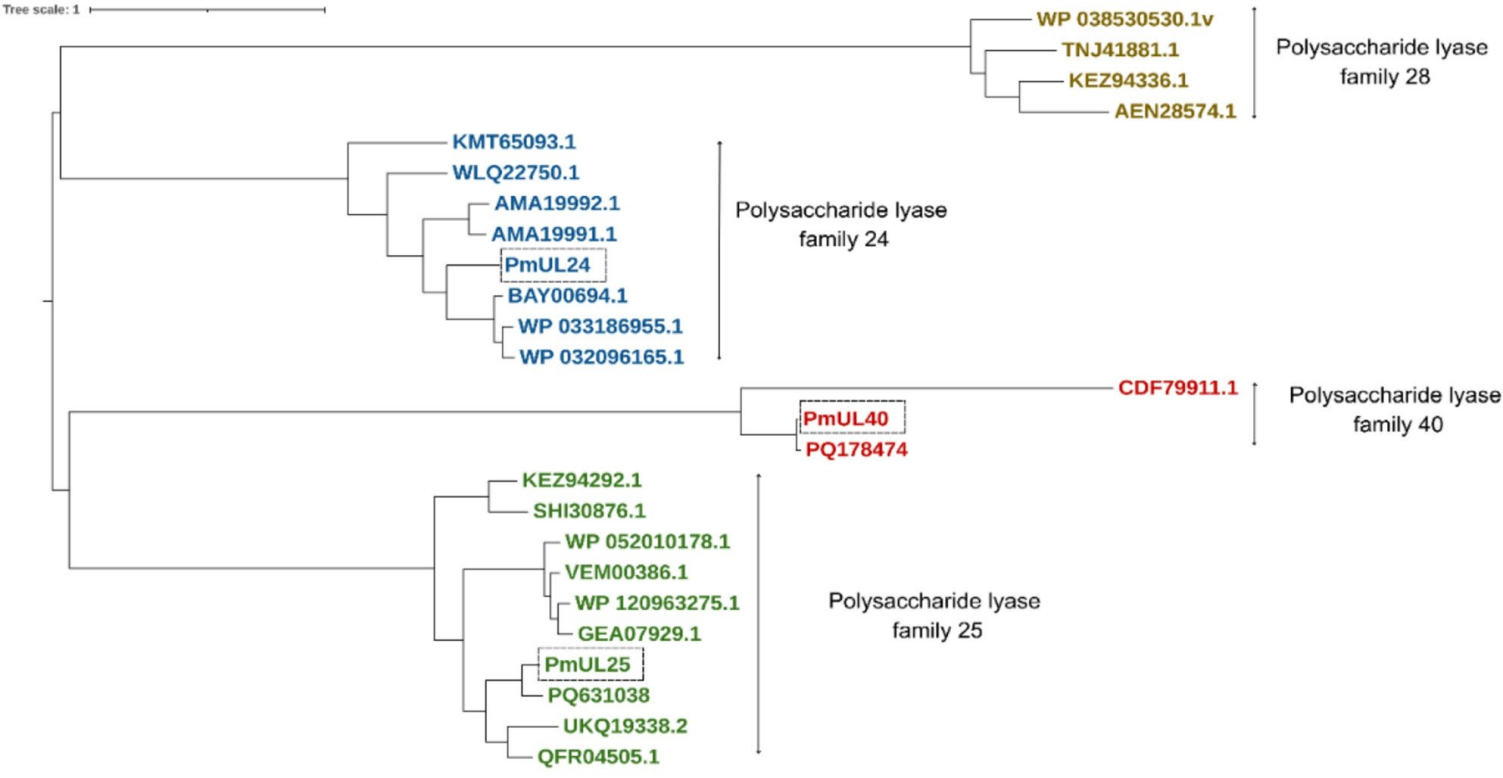
Phylogenetic analysis of polysaccharide lyase families reveals distinct evolutionary clustering of ulvan lyases. Maximum likelihood phylogenetic tree showing the evolutionary relationships among polysaccharide lyase (PL) families, with emphasis on ulvan lyase families PL24, PL25, PL28, and PL40. Characterized enzymes from different PL families were retrieved from the carbohydrate active enymes (CAZy) database. Branch colors indicate distinct PL families are shown at major nodes. The phylogenetic distribution demonstrates the divergent evolutionary origins of ulvan lyases across multiple PL families, reflecting their functional specialization in ulvan degradation

Signal peptide prediction revealed N-terminal secretion signals in all three ulvan lyases: 29 amino acids in PmUL24, 21 amino acids in PmUL25, and 40 amino acids in PmUL40. Domain architecture analysis indicated that none of the ulvan lyases contain transmembrane regions, consistent with their predicted extracellular localization (Supplementary Fig. 2). The calculated theoretical molecular weights and isoelectric points for the full-length proteins are 117.31 kDa/pI 5.13 (PmUL24), 53.01 kDa/pI 8.13 (PmUL25), and 96.67 kDa/pI 7.95 (PmUL40). Sequence similarity searches revealed that PmUL24 shares 69.43% amino acid identity with an ulvan lyase from *Alteromonas* sp. KUL17 (BAY00694.1), PmUL25 exhibits 86.11% identity to a characterized enzyme from *Pseudoalteromonas agarivorans* PUA1002 (PQ631038), and PmUL40 shows 97.54% identity to another *P. agarivorans* PUA1002 enzyme (PQ178474). Phylogenetic analysis clearly demonstrated that PmUL24, PmUL25, and PmUL40 cluster in distinct clades corresponding to their respective PL families, confirming their classification and evolutionary divergence among ulvan lyase subfamilies (Fig. 1). All the identified ulvan lyases are deposited in the NCBI databank under accession numbers: PX584653 (PmUL24), PX584654 (PmUL25), and PX584655 (PmUL40).

### Cloning, overexpression, and purification of recombinant proteins

All His-tag fused ulvan lyases were successfully expressed as soluble proteins in *E. coli* BL21(DE3). Following purification, the protein concentrations were determined to be 1.06 mg/mL for PmUL24, 9.87 mg/mL for PmUL25, and 8.01 mg/mL for PmUL40. SDS-PAGE analysis (Supplementary Fig. 3) confirmed the purity of the proteins and revealed apparent molecular weights of 120 kDa for PmUL24, 55 kDa for PmUL25, and 100 kDa for PmUL40, which closely align with their respective theoretical molecular weights. The yield of soluble PmUL24 protein was notably lower compared to the other ulvan lyases.

### Biochemical characterization of ulvan lyases

**Fig. 2:**
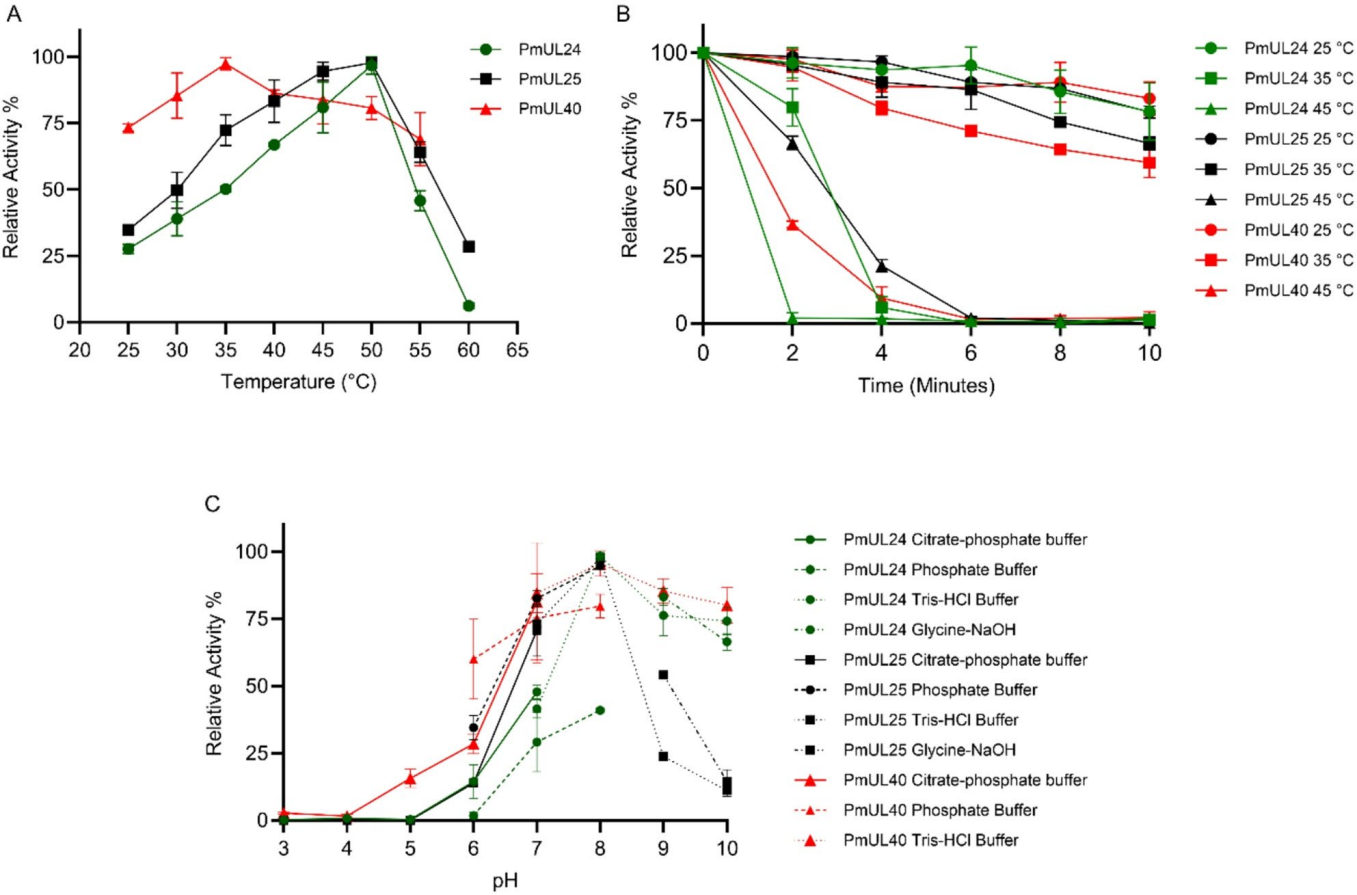
Biochemical characterization of recombinant ulvan lyases. **A** Temperature optimization showing the relative activity of each enzyme across a temperature range. **B** Thermal stability profiles indicating residual activity after pre-incubation at different temperatures. **C** pH optimization demonstrating the effect of pH on enzyme activity in different pH buffers. Green: PmUL24; black: PmUL25; red: PmUL40. Data points represent mean values ± standard deviation from three independent experiments. PmUL24 and PmUL25 exhibited optimal activity at 50 °C, while PmUL40 showed maximum activity at 35 °C. All three enzymes displayed highest activity at pH 8.0, 50mM Tris-HCl

**Fig. 3:**
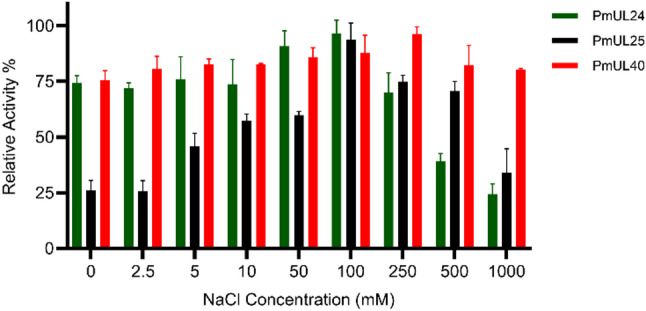
Effect of NaCl concentration on ulvan lyase activity. Relative activity of recombinant ulvan lyases measured across NaCl concentrations ranging from 0 to 1000 mM. Green: PmUL24; black: PmUL25; red: PmUL40. Data points represent mean values ± standard deviation from three independent experiments. PmUL24 and PmUL25 exhibited maximum activity at 100 mM NaCl, while PmUL40 showed optimal activity at 250 mM NaCl

The biochemical characterization of purified ulvan lyases revealed distinct temperature profiles, with both PmUL24 and PmUL25 exhibiting maximum activity at 50 °C, while PmUL40 showed the highest activity at 35 °C (Fig. 2A). Among the three enzymes, thermal stability profiles were similar, with PmUL25 and PmUL40 exhibiting comparatively higher stability than PmUL24 (Fig. 2B). Regarding pH optimization, all three ulvan lyases displayed maximum activity at pH 8.0 in 50 mM Tris-HCl buffer, indicating a preference for slightly alkaline conditions (Fig. 3C). Generally, ulvan lyases function in marine environments, and the influence of NaCl concentration is a critical parameter in their biochemical characterization (Fig. 3). PmUL24 and PmUL25 showed peak activities at 100 mM NaCl, following similar trends but with notable differences in salt tolerance. PmUL24 activity decreased rapidly beyond the optimal concentration, retaining only 25% activity at 1 M NaCl, whereas PmUL25 demonstrated greater salt tolerance, exhibiting a gradual decline in activity up to 1 M NaCl. PmUL40 displayed the highest NaCl tolerance among the three enzymes, with maximum activity at 250 mM NaCl.

**Fig. 4:**
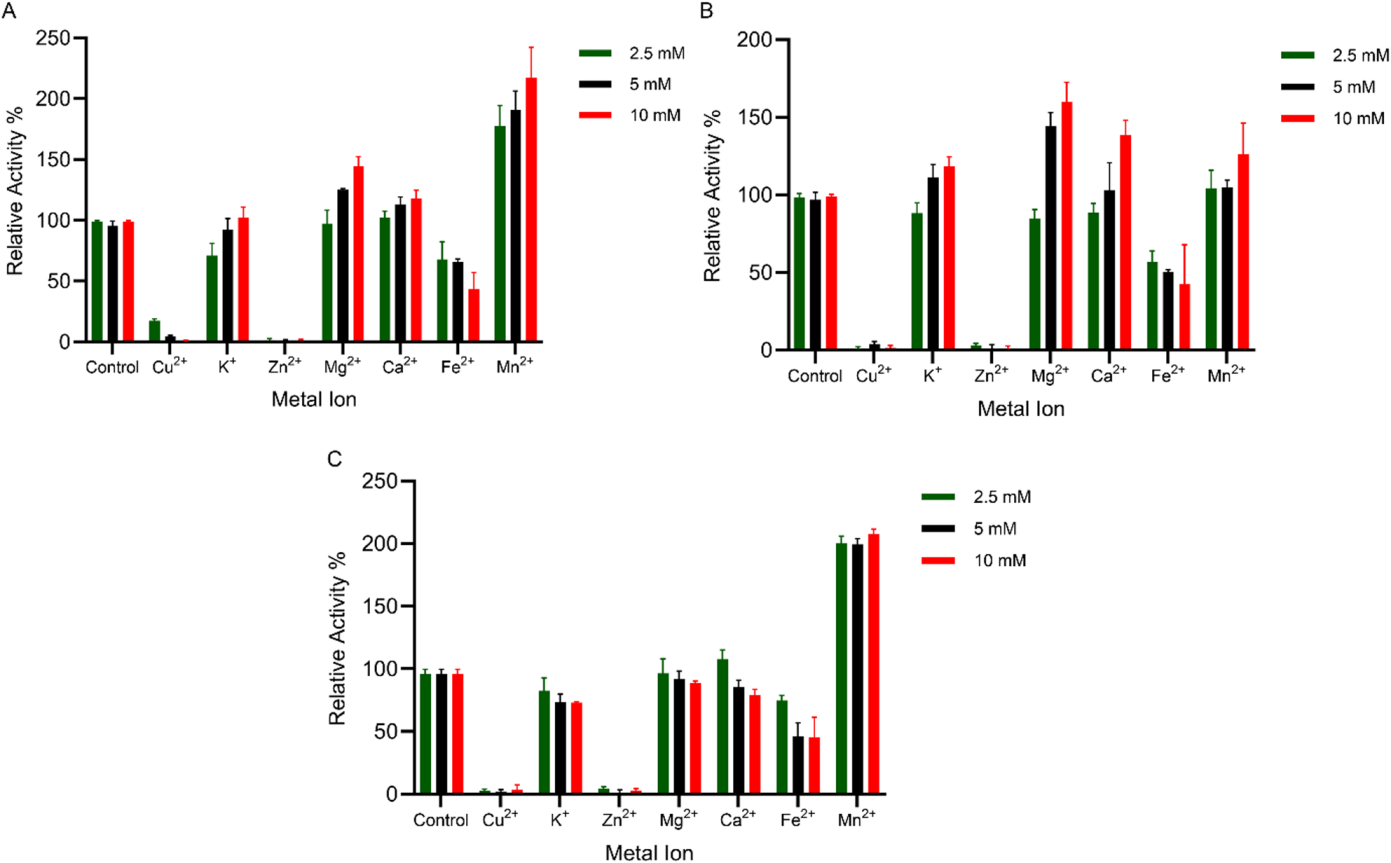
Effect of metal ions on ulvan lyase activity. Relative activity of recombinant ulvan lyases in the presence of various metal ions at different concentrations. **A** PmUL24, **B** PmUL25, and **C** PmUL40. Green bars: 2.5 mM; black bars: 5 mM; red bars: 10 mM metal ion concentration. Data represent mean values ± standard deviation from three independent experiments

Metal ion interactions significantly influenced ulvan lyase activity, with distinct patterns observed for each enzyme. PmUL24 showed enhanced activity in the presence of K⁺, Mg²⁺, Ca²⁺, and Mn²⁺ at concentrations up to 10 mM (Fig. 4A) while Fe²⁺ progressively reduced enzyme activity with increasing concentration, and Cu²⁺ and Zn²⁺ completely abolished enzymatic function. PmUL25 exhibited a metal ion interaction profile similar to PmUL24 (Fig. 4B), with K⁺, Mg²⁺, Ca²⁺, and Mn²⁺ enhancing enzyme activity, whereas Fe²⁺ was the only metal ion that reduced activity, and Cu²⁺ and Zn²⁺ caused protein denaturation and complete loss of function, even at low concentrations (2.5 mM). PmUL40 displayed a markedly different metal ion interaction pattern compared to the other two ulvan lyases, with Mn²⁺ being the only metal ion that positively influenced enzyme activity, while the other examined metal ions (K⁺, Mg²⁺, Ca²⁺, and Fe²⁺) decreased activity with increasing concentration (Fig. 4C). Similar to PmUL24 and PmUL25, Cu²⁺ and Zn²⁺ completely deactivated PmUL40. Among all tested metal ions, Mn²⁺ was the only ion that consistently enhanced the activity of all three ulvan lyases, producing approximately 2-fold enhancement for PmUL24 and PmUL40, and 1.25-fold enhancement for PmUL25.

The chromatograms displayed distinct separation of ulvan depolymerization products (Supplementary Fig. 4). PmUL24 predominantly produced DP2(ΔUA-R3S) as the major product, with minor amounts of DP2(G-R3S). PmUL25 also generated DP2(ΔUA-R3S) as the primary product, but uniquely produced DP4(ΔUA-R3S; X-R3S) tetramers in addition to the disaccharide products. PmUL40 exhibited a product profile similar to PmUL24, yielding DP2(ΔUA-R3S) as the dominant product along with smaller quantities of DP2(G-R3S). Overall, all three enzymes produced DP2 oligomers as their main depolymerization products, while PmUL25 was distinguished by its ability to generate DP4 oligomers alongside the disaccharides.

### Ulvan lyases structural analysis

According to the SWISS-MODEL results, PmUL24 showed a QMEANDisCo Global value of 0.85 ± 0.05 with a sequence identity of 69.42% to the template, while PmUL25 obtained a QMEANDisCo Global value of 0.83 ± 0.05 with a sequence identity of 61.45%. QMEANDisCo values approaching 1.0 indicate high-quality models; therefore, these quality metrics confirmed that the predicted structures were reliable for subsequent molecular docking studies.

Multiple sequence alignment of PmUL24 with other characterized ulvan lyases revealed that residues His135, His156, Tyr228, and Arg244 might be key residues for catalytic function. Similarly, His106, His126, Tyr171, and Arg187 were identified as potentially critical residues for the catalytic activity of PmUL25 (Supplementary Fig. 5). Since these results are based on computational predictions, the actual key residues may differ slightly under experimental conditions [37, 38]. However, these predictions were supported by multiple bioinformatic approaches. Molecular docking was subsequently conducted to further validate whether the aforementioned key residues could interact with ulvan oligomer ligands.

Molecular docking simulations were performed to predict the substrate binding and degradation mechanism using homology modeled protein structures (PmUL24 and PmUL25) and the ulvan DP4 ligand (ΔUA-GlcA-Rha3S-GlcA-Rha3S). The binding affinity between PmUL24 and the DP4 ligand was − 8.8 kcal/mol, while PmUL25 showed a binding affinity of − 7.8 kcal/mol with the DP4 ligand. According to the literature, ulvan degradation proceeds via a β-elimination reaction following substrate binding to the protein. His/Tyr-dependent catalytic mechanisms have been reported for the template structures PDB 5UAM and 6BYP [29, 30]. Consistent with these mechanisms, the docking simulations of both PmUL24 and PmUL25 ulvan lyases revealed similar catalytic strategies.

**Fig. 5:**
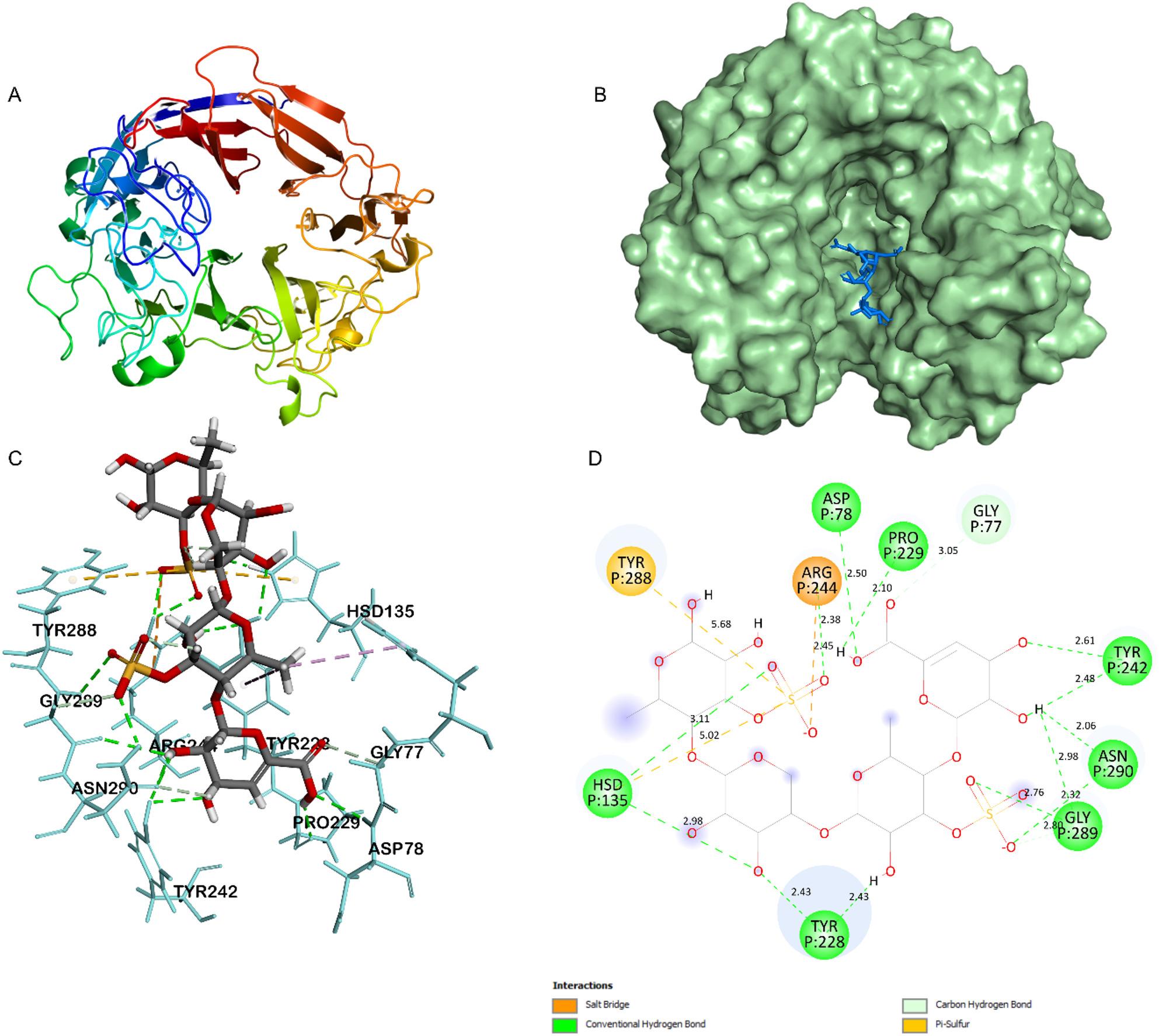
Structural analysis and molecular docking of PmUL24. **A** Overall three-dimensional structure of PmUL24 generated by homology modeling using PDB template 6BYP. **B** Molecular docking complex showing PmUL24 (surface representation) with DP4 oligosaccharide substrate (stick representation) positioned within the catalytic pocket. **C** Three-dimensional visualization of ligand-receptor interactions highlighting key residues involved in substrate binding and catalysis. **D** Two-dimensional interaction diagram illustrating the detailed molecular interactions between PmUL24 and DP4

In PmUL24, His135 is highly likely to function as the catalytic acid/base (proton abstractor). This residue forms hydrogen bonds (2.98 Å, 3.11 Å) and a π-sulfur interaction (5.02 Å) that precisely anchors the ligand for catalysis. Arg244 serves as the primary anchoring residue by forming a strong salt bridge (2.45 Å), which neutralizes the negative charge on the ulvan ligand. Tyr228 contributes two conventional hydrogen bonds (2.43 Å each) that assist in the proper alignment and orientation of the substrate for the catalytic function of His135 (Fig. 5).

**Fig. 6:**
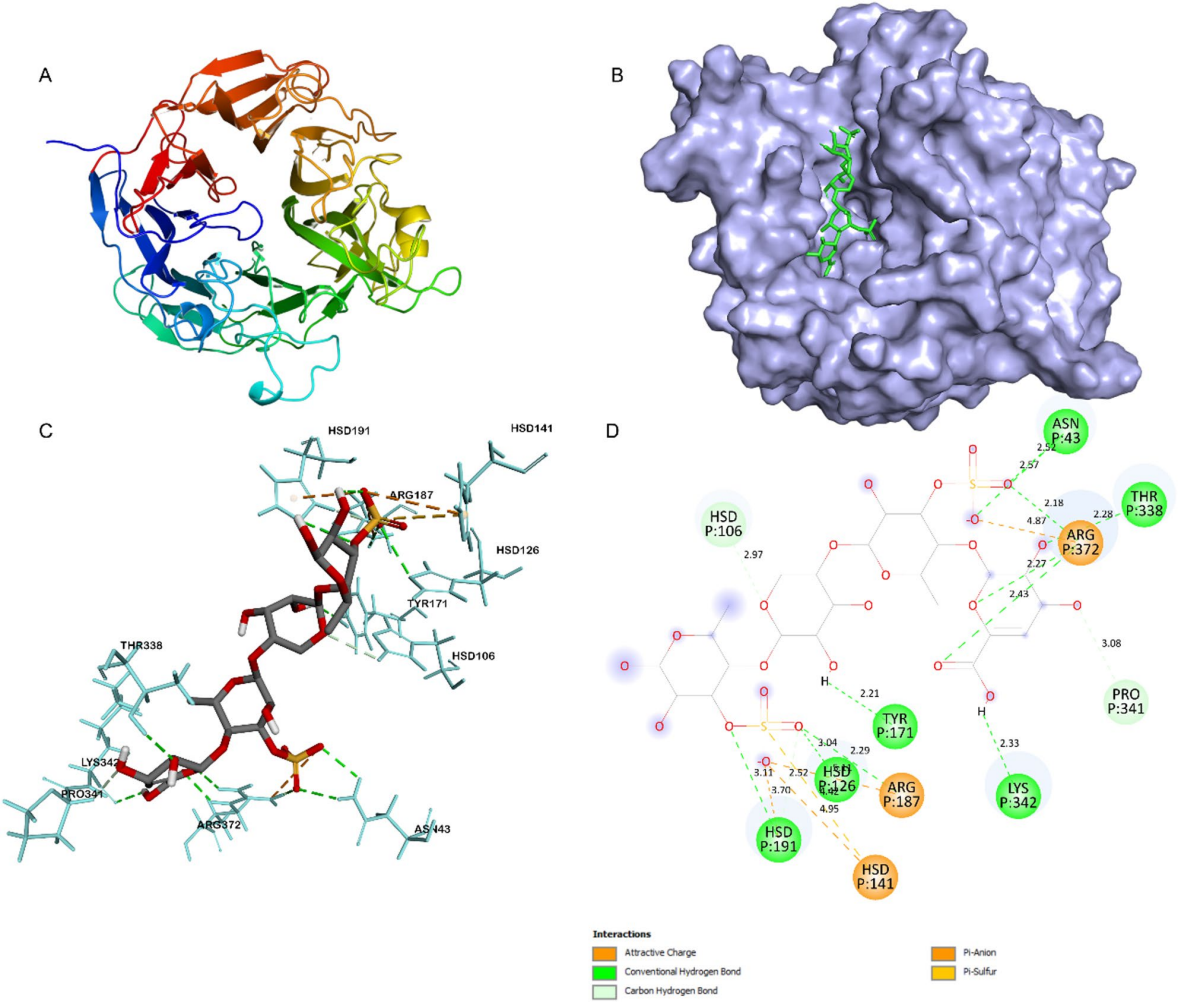
Structural analysis and molecular docking of PmUL25. **A** Overall three-dimensional structure of PmUL25 generated by homology modeling using PDB template 5UAM. **B** Molecular docking complex showing PmUL25 (surface representation) with DP4 oligosaccharide substrate (stick representation) positioned within the catalytic pocket. **C** Three-dimensional visualization of ligand-receptor interactions highlighting key residues involved in substrate binding and catalysis. **D** Two-dimensional interaction diagram illustrating the detailed molecular interactions between PmUL25 and DP4

In the PmUL25 and DP4 interaction, His126 is predicted to function as the acid/base catalytic residue, positioned near the scissile glycosidic bond with a conventional hydrogen bond of 3.04 Å. Similar to the PmUL24 interaction, Arg187 stabilizes the negative charge of the ulvan ligand through an attractive charge interaction (5.11 Å) and a hydrogen bond (2.29 Å). Tyr171 ensures proper substrate orientation for catalysis by forming a conventional hydrogen bond (2.21 Å) with GlcA. In addition to the key residues identified through multiple sequence alignment, Arg372 in PmUL25 makes strong interactions with the ligand through hydrogen bonds and attractive charge interactions, further neutralizing the overall charge of the DP4 ligand (Fig. 6).

The predicted binding modes of both PmUL24 and PmUL25 suggest that these enzymes can bind the ulvan DP4 ligand and cleave the substrate through His/Tyr-dependent catalysis, consistent with the mechanisms observed in the 5UAM and 6BYP ulvan lyase structures.

### Comparative genome analysis and prediction of ulvan utilization loci

The OrthoANI-based phylogenomic analysis illustrates the genomic relatedness between *Pseudoalteromonas marina* strain PUA1001 and other representative *Pseudoalteromonas* species (Supplementary Fig. 6). The dendrogram displays average nucleotide identity (ANI) values ranging from 81% to 100% on the x-axis. Strain PUA1001 clustered most closely with *P. marina* strain mano4, exhibiting an OrthoANI value of approximately 97.5%, which exceeds the species delineation threshold of 95–96% ANI. This high genomic similarity confirms the species-level identification of PUA1001 as *P. marina*. In contrast, PUA1001 showed substantially lower ANI values (~ 84%) with other *Pseudoalteromonas* species, including *P. carrageenovora* ATCC 43,555, *P. arctica* A 37-1-2T, and *P. distincta* ATCC 700518T, confirming clear taxonomic separation at the species level.

The POM visualizes orthologous relationships between all CDSs of *P. marina* PUA1001 and five representative *Pseudoalteromonas* genomes (Supplementary Table 1). The heatmap color scheme represents sequence identity levels of reciprocal best BLAST hits, ranging from high similarity (dark blue) to low or absent orthology (red). Genome-wide comparison revealed that PUA1001 shares extensive orthology with *P. marina* mano4, as evidenced by the predominance of dark blue and cyan cells, consistent with their high OrthoANI value (97.5%) and conspecific relationship. Orthologous gene content progressively declined across more distantly related species (*P. arctica*,* P. carrageenovora*,* and P. distincta*), manifested as decreasing color intensity in the matrix. This gradient reflects the phylogenetic distance from the *P. marina* lineage. A distinct pattern emerged for contig 3, where the majority of genes showed weak or absent orthology (red coloration) across all compared genomes, including the closely related *P. marina* mano4. This concentration of singleton genes suggests that contig 3 harbors strain-specific genetic elements, potentially including acquired genes involved in specialized metabolic capabilities or ecological adaptations.

Venn diagram analysis delineated the pan genome structure across five *Pseudoalteromonas* genomes (Supplementary Fig. 7). The core genome comprised 2,924 orthologous gene clusters present in all strains, encompassing essential cellular processes and conserved metabolic pathways characteristic of the genus. The accessory genome of *P. marina* PUA1001 included 275 strain-specific gene clusters with no detectable orthologs in the compared genomes. Functional predictions indicate these unique genes likely contribute to strain-specific phenotypes, including adaptation to particular ecological niches and metabolism of specialized polysaccharides such as ulvan. Each of the other examined strains similarly possessed unique gene sets, consistent with their independent evolutionary histories and ecological specialization. Contig 3 exhibited a remarkable concentration of strain-specific genes: 74 of 78 total genes (94.9%) showed less than 90% sequence identity to any ortholog in the compared genomes (Supplementary Fig. 8). This high proportion of unique genes, far exceeding the genome-wide average, raises the possibility that contig 3 represents a horizontally acquired genomic island encoding novel metabolic capabilities specific to PUA1001.

Detailed functional annotation of contig 3 CDSs revealed a distinct polysaccharide utilization system absent from *P. marina* mano4. The proposed locus encodes three ulvan lyases belonging to polysaccharide lyase (PL) families 24, 25, and 40, which catalyze the depolymerization of ulvan through β-elimination. The genetic organization suggests a complete catabolic pathway for ulvan degradation, encompassing: (i) degradative enzymes, including glycoside hydrolases and sulfatases for glycosidic bond cleavage and desulfation; (ii) metabolic enzymes such as sugar isomerases and disaccharide hydrolases for processing released monosaccharides and disaccharides; and (iii) genes involved in L-rhamnose metabolism, a major component of ulvan. The locus also contains genes encoding substrate recognition and transport machinery, including a TonB-dependent receptor for polysaccharide binding and uptake across the outer membrane, Tripartite ATP-independent periplasmic (TRAP) transporters, and MFS (major facilitator superfamily) transporters for sugar internalization. This genetic architecture is characteristic of PULs commonly found in marine bacteria specialized for complex carbohydrate degradation.

**Fig. 7:**
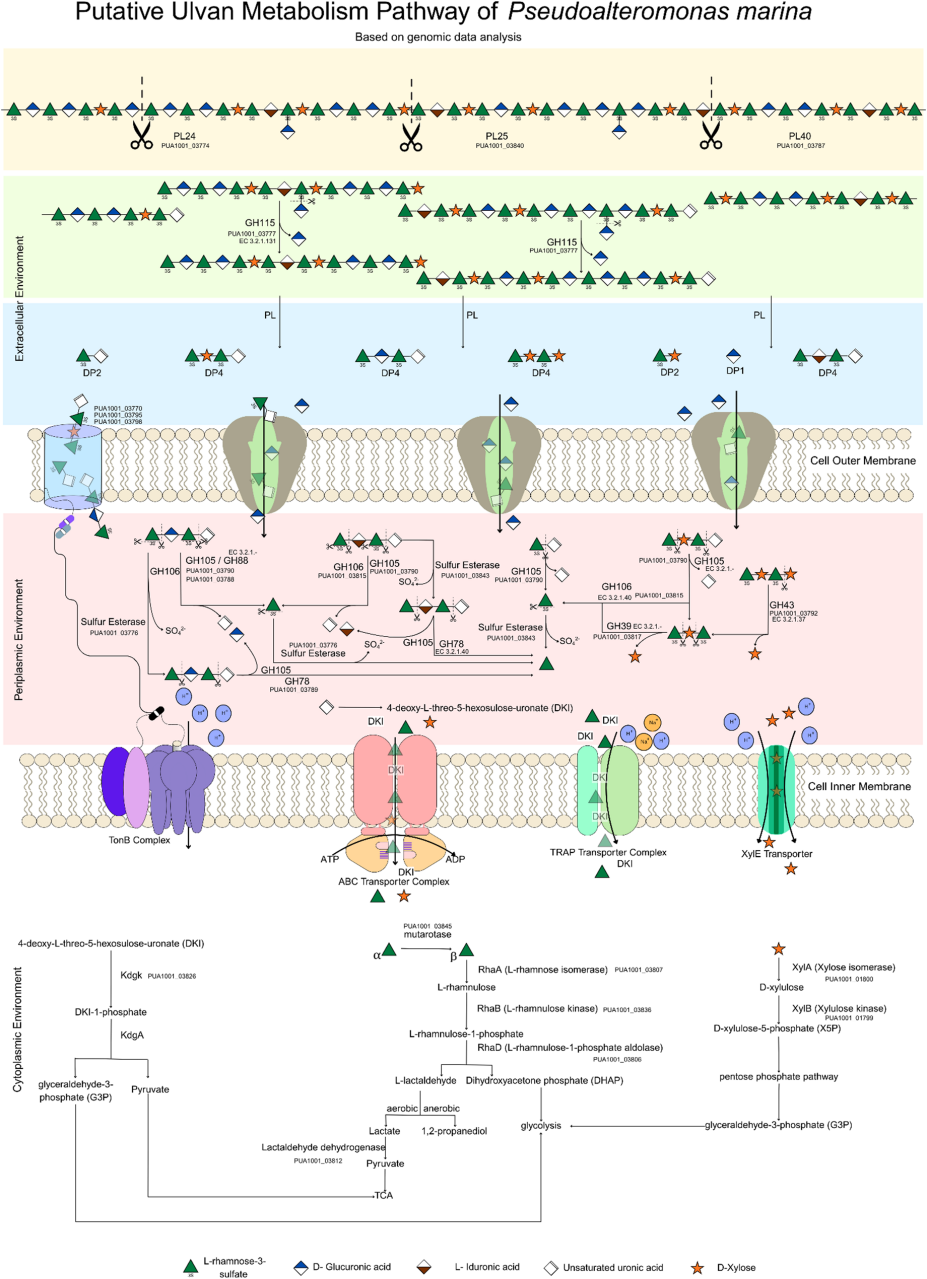
Proposed ulvan degradation pathway in *Pseudoalteromonas marina* from the extracellular to cytoplasmic environment. Schematic representation of the proposed ulvan utilization system based on genomic analysis of contig 3. The pathway illustrates the sequential process of ulvan degradation, beginning with extracellular depolymerization by secreted ulvan lyases (PmUL24, PmUL25, PmUL40), followed by oligosaccharide transport across the outer membrane via TonB-dependent transporters (TBDT) and inner membrane transport through specific ATP-binding cassette (ABC) transporters or Tripartite ATP-independent periplasmic (TRAP) transporter. Within the periplasmic and cytoplasmic environments, oligosaccharides undergo further processing by intracellular glycoside hydrolases and sulfatases to release monosaccharides and sulfate groups. Arrows indicate the direction of substrate flow and enzymatic conversions. Gene designations correspond to annotations from contig 3 of the *P. marina* genome, demonstrating the organization of the ulvan utilization gene cluster and the coordinated modeled metabolic pathway for ulvan depolymerization

Based on integrative analysis of subcellular localization predictions, domain architecture, NCBI BLAST information, and biochemical characterization of recombinant enzymes, we propose a collective extracellular to intracellular ulvan degradation pathway (Fig. 7, Supplementary Table 2). Three endo-acting ulvan lyases, PL24 (PUA1001_03774), PL25 (PUA1001_03840), and PL40 (PUA1001_03787), initiate ulvan degradation in the extracellular environment. All three enzymes possess Sec/SPI signal peptides and are predicted to be secreted via the general secretory pathway. LC-MS analysis of recombinant enzyme reaction products confirmed that these lyases generate oligosaccharides with degrees of polymerization of 2 and 4 (DP2 and DP4) through β-eliminative cleavage of glycosidic bonds, producing unsaturated uronic acid residues (Δ4,5-unsaturated) at non-reducing termini. Branch chain hydrolysis of α-1,2-linked D-glucuronic acid residues from L-rhamnose in ulvan side chains is catalyzed by an extracellular glucuronyl hydrolase belonging to glycoside hydrolase family 115 (GH115; PUA1001_03777), releasing free D-glucuronic acid into the surrounding medium. Ulvan-derived oligosaccharides (DP2-DP4) are transported across the outer membrane, likely via the TonB-dependent receptor system. In the periplasm, two exo-acting glucuronyl hydrolases, GH88 (PUA1001_03788) and GH105 (PUA1001_03790), remove the terminal Δ4,5-unsaturated uronic acid residues generated by lyase activity, producing saturated oligosaccharides suitable for further catabolism.

The periplasmic space serves as the primary site for converting ulvan oligosaccharides into monosaccharides suitable for cytoplasmic transport. Following outer membrane translocation, oligosaccharides undergo extensive enzymatic modification by periplasmic enzymes. Desulfation represents a critical initial step, as sulfate groups at the C-3 positions of L-rhamnose residues must be removed before efficient glycosidic bond cleavage can occur. Two sulfatases, PUA1001_03776 and PUA1001_03843, catalyze the hydrolysis of these sulfate esters, generating desulfated substrates for downstream processing. Periplasmic glycoside hydrolases complete the depolymerization process. GH43 (PUA1001_03792) and GH39 (PUA1001_03817) β-xylosidase remove terminal xylose residues from oligosaccharide non-reducing ends, while a GH78 α-L-rhamnosidase cleaves terminal α-L-rhamnose from disaccharides, liberating free rhamnose monomers. The collective action of these enzymes converts ulvan oligosaccharides into monosaccharides.

TRAP transporters import uronic acids, MFS transporters handle rhamnose and uronic acids, XylE transports xylose, and ABC transporters provide ATP-dependent sugar import. Once internalized, monosaccharides enter specialized catabolic pathways. L-rhamnose is converted to dihydroxyacetone phosphate and pyruvate via the rhamnose pathway. Uronic acids are processed through uronate pathways to yield pyruvate and glyceraldehyde-3-phosphate. D-xylose enters the pentose phosphate pathway as xylulose-5-phosphate. These pathways are predicted to interface with glycolysis and the Krebs cycle, indicating a potential metabolic route through which ulvan-derived carbon could contribute to central metabolism and biosynthetic processes in marine bacteria.

## Discussion

Ulvan depolymerization by marine bacteria is a complex process requiring specialized enzymes and specific environmental conditions. To date, only a limited number of marine microorganisms have been documented with ulvan-degrading capability. Among characterized ulvan degraders, the genus *Alteromonas* and *Pseudoalteromonas* are the most frequently reported. Historically, *Pseudoalteromonas* species were classified within the genus *Alteromonas*, but were later separated into a distinct genus based on phylogenetic, genetic, and physiological characteristics [39]. *Pseudoalteromonas* species represent one of the most abundant heterotrophic bacterial groups in marine ecosystems, exhibiting remarkable ecological versatility. They have been isolated from diverse marine habitats, including seawater, marine sediments, invertebrate hosts, and macroalgal surfaces, reflecting their adaptability to various ecological niches and their potential roles in marine carbon cycling and polysaccharide degradation [40, 41].

The ulvan utilization locus characterized in *F. agariphila* KMM 3901 represents the most extensively documented system for ulvan degradation to date. The ulvan utilization system identified in *P. marina* PUA1001 exhibits structural and functional similarities to this reference locus. However, polysaccharide metabolism in *Pseudoalteromonas* species, and particularly in *P. marina* PUA1001, remains poorly characterized in the literature. A key distinction between these ulvan-degrading organisms lies in their taxonomic classification and associated metabolic strategies. *F. agariphila* KMM 3901 belongs to the phylum Bacteroidota, while *P. marina* PUA1001 is a member of the phylum Pseudomonadota (https://www.ncbi.nlm.nih.gov/taxonomy). These phyla exhibit fundamentally different approaches to carbohydrate metabolism and occupy distinct ecological roles in marine ecosystems. Members of Bacteroidota are recognized as specialized complex polysaccharide degraders, with genomes typically enriched in CAZymes organized into polysaccharide utilization loci [42]. This genomic architecture reflects their evolutionary adaptation as primary degraders of complex polysaccharides in marine environments. In contrast, Pseudomonadota have traditionally been characterized as opportunistic heterotrophs with more generalized metabolic capabilities and comparatively fewer CAZymes [43, 44].

However, the presence of predicted ulvan degradation systems in *Pseudoalteromonas* species is a notable metabolic expansion beyond the typical phylum Pseudomonadota repertoire. This acquisition of specialized polysaccharide-degrading machinery may reflect horizontal gene transfer from Bacteroidota or other polysaccharide-degrading taxa, potentially contributing to the ability of *Pseudoalteromonas* species to utilize ulvan-rich niches associated with green macroalgae. The metabolic versatility conferred by such polysaccharide utilization systems likely contributes to the ecological success and widespread distribution of *Pseudoalteromonas* species in diverse marine habitats, allowing them to thrive in environments where algal polysaccharides represent a significant carbon source. Furthermore, comparative analysis of the PUA1001 ulvan utilization locus related to *F. agariphila* KMM 3901 revealed the presence of two additional glycoside hydrolase families: GH106 and GH115 that have not been reported in previously characterized ulvan degradation systems. According to the CAZy database, GH106 family enzymes typically exhibit α-L-rhamnosidase activity, while GH115 family members function as α-glucuronidases with specificity for α-1,2-glycosidic linkages. The presence of GH115 is particularly significant, as it enables hydrolytic removal of α-1,2-linked D-glucuronic acid branches from the ulvan backbone, complementing the debranching activities of other enzymes in the locus. The inclusion of both GH106 and GH115 suggests that the PUA1001 system possesses enhanced capability for processing structurally complex ulvan variants, potentially providing a more comprehensive putative degradation pathway compared to previously documented systems. Notably, comparative genomic analysis revealed that the closely related strain *P. marina* mano4, which shares 97.5% OrthoANI with PUA1001, completely lacks the ulvan utilization locus and associated genes (Supplementary Table 1). This prominent absence of ulvan-degrading capability in mano4, despite high overall genomic similarity, provides evidence for strain-level metabolic specialization within the same species. The presence or absence of this modeled catabolic pathway between conspecific strains suggests that the ulvan utilization locus in PUA1001 is strain specific and may have been acquired through horizontal gene transfer, although alternative scenarios such as vertical inheritance followed by lineage-specific gene loss cannot be excluded. Furthermore, ulvan-degrading enzymes are co-localized within a single genomic region on contig 3, forming a discrete gene cluster characteristic of polysaccharide utilization loci. This syntenic organization is characteristic of polysaccharide utilization loci and is consistent with the potential for coordinated regulation and functional integration of the encoded enzymes. Additionally, this genomic flexibility highlights the role of accessory genes in driving ecological differentiation and niche adaptation among closely related bacterial strains. The ulvan-degrading capability may confer an ecological advantage to PUA1001 in environments enriched in *Ulva* biomass, whereas mano4 may have adapted to alternative ecological niches or carbon sources.

The biochemical characterization of ulvan lyases revealed that PmUL24, PmUL25, and PmUL40 function within the typical ranges documented for characterized ulvan lyases in terms of temperature optima, pH preferences, and thermal stability. Most reported ulvan lyases exhibit optimal activity at temperatures between 35 and 60 °C, pH ranges of 8–10, and show reduced thermal stability after incubation above 30 °C for extended periods (> 1 h) [45]. For example, PL24 family members such as AMA19992.1 show optimal activity at 50 °C and pH 8.0 [46], while KMT65093.1 functions optimally at 40 °C and pH 9.0 [47]. Similarly, PL25 family enzymes, including UKQ19338.1, display peak activity at 60 °C and pH 9.0 [48], whereas PQ631038 exhibits optimal function at 35 °C and pH 10.0 [49]. The PL40 family representative PQ178474 shows maximum activity at 35 °C and pH 8.0, consistent with the properties observed for PmUL40 in this study [19]. Metal ion effects also follow established patterns, with Cu²⁺ and Zn²⁺ demonstrating inhibitory effects on most ulvan lyases across different families. The role of Mn²⁺ has been more variable in previous reports, showing activating effects on some ulvan lyases while inhibiting others [45]. Notably, the ulvan lyases characterized in this study displayed significant activation by Mn²⁺, with a 2-fold enhancement observed for PmUL24 and PmUL40, and 1.25-fold enhancement for PmUL25, suggesting that Mn²⁺ plays a critical role in the catalytic mechanism of these enzymes. Molecular docking for PmUL40 was excluded due to the current absence of experimentally determined crystal structures within the PL40 family. However, our molecular docking results for PmUL24 and PmUL25 align with the established His/Tyr-dependent β-elimination mechanism reported for structurally characterized ulvan lyases from families PL24 and PL25. In both PmUL24 and PmUL25, histidine and tyrosine residues formed critical interactions with the ulvan DP4 substrate, suggesting conservation of this catalytic strategy across ulvan-degrading enzymes [29, 30]. These findings are consistent with the notion that ulvan lyases from marine bacteria share conserved biochemical properties that are compatible with marine environmental conditions.

## Conclusions

This study provides a comprehensive biochemical characterization of three novel ulvan lyases from the marine bacterium *Pseudoalteromonas marina* PUA1001, isolated from Jeju Island, South Korea, and proposes a putative ulvan utilization pathway representative of the phylum Pseudomonadota. The characterized ulvan lyases belong to three distinct polysaccharide lyase families: PmUL24 (PL24), PmUL25 (PL25), and PmUL40 (PL40), and predominantly depolymerize ulvan into DP2 oligomers, with PmUL25 additionally producing minor amounts of DP4 products. The elucidated modeled ulvan utilization pathway demonstrates how marine bacteria have evolved specialized enzymatic machinery for efficient polysaccharide degradation, reflecting their adaptive strategies for thriving in diverse ecological niches within marine ecosystems.

## Supplementary Information

Below is the link to the electronic supplementary material.

Supplementary Material 1.

Supplementary Material 2.

Supplementary Material 3.

Supplementary Material 4.

## Data Availability

The data underlying this article are available in the article and in its online supplementary material.
