# Supplementary material for: Biocatalytic ulvan degradation by Pseudoalteromonas marina: exploring a marine polysaccharide bioconversion system
Source: Microb Cell Fact. 2026 Feb 27;25:85. doi: 10.1186/s12934-026-02969-0 (PMC13050008; doi:10.1186/s12934-026-02969-0)
Supplement: Supplementary file 1 — Supplementary Material 1. [file 12934_2026_2969_MOESM1_ESM.docx]

**Biocatalytic Ulvan Degradation by Pseudoalteromonas marina: Exploring a Marine Polysaccharide Bioconversion system**

Navindu Dinara Gajanayaka,^a,b^ Eunyoung Jo,^a^ Minthari Sakethanika Bandara,^a,b^ Jaewon Lee,^a,b^ Svini Dileepa Marasinghe,^a^ Jonathan Sathyadith,^a,b^ Tae-Yang Eom,^a^ Gun-Hoo Park,^a,b^ Chulhong Oh,^a,b,#^ Youngdeuk Lee^a,#^

^a^Jeju Bio Research Center, Korea Institute of Ocean Science and Technology (KIOST), Jeju-si, Republic of Korea.

^b^ Department of Marine Technology & Convergence Engineering (Marine Biotechnology), KIOST School, Korea National University of Science and Technology, Daejeon, Republic of Korea.

Keywords: Ulvan depolymerization, Ulvan utilization pathway, Ulvan lyases, *Pseudoalteromonas marina*, Recombinant protein expression

^#^Address correspondence to:

Chulhong Oh, Ph.D.: Mailing address: Jeju Bio Research Center, Korea Institute of Ocean Science and Technology (KIOST), 2670 Ilju-dong-ro, Gujwa-eup, Jeju-si 63349, Republic of Korea; Phone: (82) 64 798 6102; Fax: (82) 64 798 6039; E-mail: [och0101@kiost.ac.kr](mailto:och0101@kiost.ac.kr)

Youngdeuk Lee, Ph.D.: Mailing address: Jeju Bio Research Center, Korea Institute of Ocean Science and Technology (KIOST), 2670 Ilju-dong-ro,Gujwa-eup, Jeju-si 63349, Republic of Korea; Phone: (82)64 798 6105; Fax: (82) 64 798 6191; E-mail: [lyd1981@kiost.ac.kr](mailto:lyd1981@kiost.ac.kr)

Supplementary Table 1 Tab 1: Pairwise ortholog matrix comparing complete draft genomes across *Pseudoalteromonas* species. Shading intensity represents the degree of ortholog conservation: ≥99 %, ≥95 %, ≥90 %, <90 %, No-reciprocal, and No hit. Tab 2: Pairwise ortholog matrix for genes located on contig 3 across *Pseudoalteromonas* genomes. Color coding indicates sequence similarity levels and ortholog presence as defined above.

Supplementary Table 2 Tab 1: Predicted subcellular localization of proteins using multiple web-based prediction tools. Tab 2: Consensus subcellular localization assignments based on integrated computational analysis and NCBI BLAST results showing the top match for each protein. Tab 3: Additional genes supporting the ulvan utilization locus identified outside of contig 3, with corresponding NCBI BLAST top hits.
