# Supplementary material for: Biocatalytic ulvan degradation by Pseudoalteromonas marina: exploring a marine polysaccharide bioconversion system
Source: Microb Cell Fact. 2026 Feb 27;25:85. doi: 10.1186/s12934-026-02969-0 (PMC13050008; doi:10.1186/s12934-026-02969-0)
Supplement: Supplementary file 4 — Supplementary Material 4. [file 12934_2026_2969_MOESM4_ESM.docx]

**Biocatalytic Ulvan Degradation by Pseudoalteromonas marina: Exploring a Marine Polysaccharide Bioconversion system**

Navindu Dinara Gajanayaka,^a,b^ Eunyoung Jo,^a^ Minthari Sakethanika Bandara,^a,b^ Jaewon Lee,^a,b^ Svini Dileepa Marasinghe,^a^ Jonathan Sathyadith,^a,b^ Tae-Yang Eom,^a^ Gun-Hoo Park,^a,b^ Chulhong Oh,^a,b,#^ Youngdeuk Lee^a,#^

^a^Jeju Bio Research Center, Korea Institute of Ocean Science and Technology (KIOST), Jeju-si, Republic of Korea.

^b^ Department of Marine Technology & Convergence Engineering (Marine Biotechnology), KIOST School, Korea National University of Science and Technology, Daejeon, Republic of Korea.

Keywords: Ulvan depolymerization, Ulvan utilization pathway, Ulvan lyases, *Pseudoalteromonas marina*, Recombinant protein expression

^#^Address correspondence to:

Chulhong Oh, Ph.D.: Mailing address: Jeju Bio Research Center, Korea Institute of Ocean Science and Technology (KIOST), 2670 Ilju-dong-ro, Gujwa-eup, Jeju-si 63349, Republic of Korea; Phone: (82) 64 798 6102; Fax: (82) 64 798 6039; E-mail: [och0101@kiost.ac.kr](mailto:och0101@kiost.ac.kr)

Youngdeuk Lee, Ph.D.: Mailing address: Jeju Bio Research Center, Korea Institute of Ocean Science and Technology (KIOST), 2670 Ilju-dong-ro,Gujwa-eup, Jeju-si 63349, Republic of Korea; Phone: (82)64 798 6105; Fax: (82) 64 798 6191; E-mail: [lyd1981@kiost.ac.kr](mailto:lyd1981@kiost.ac.kr)

Supplementary Figure 1: Ulvan lyase activity and colony morphology of Pseudoalteromonas marina PUA1001. (a) Ulvan-degrading activity of P. marina PUA1001 showing a clear halo zone on a 10% ulvan–seawater agar plate after incubation at 25 °C for 2–3 days. (b)Colony morphology of P. marina PUA1001 grown on a marine agar plate after overnight incubation at 30 °C.


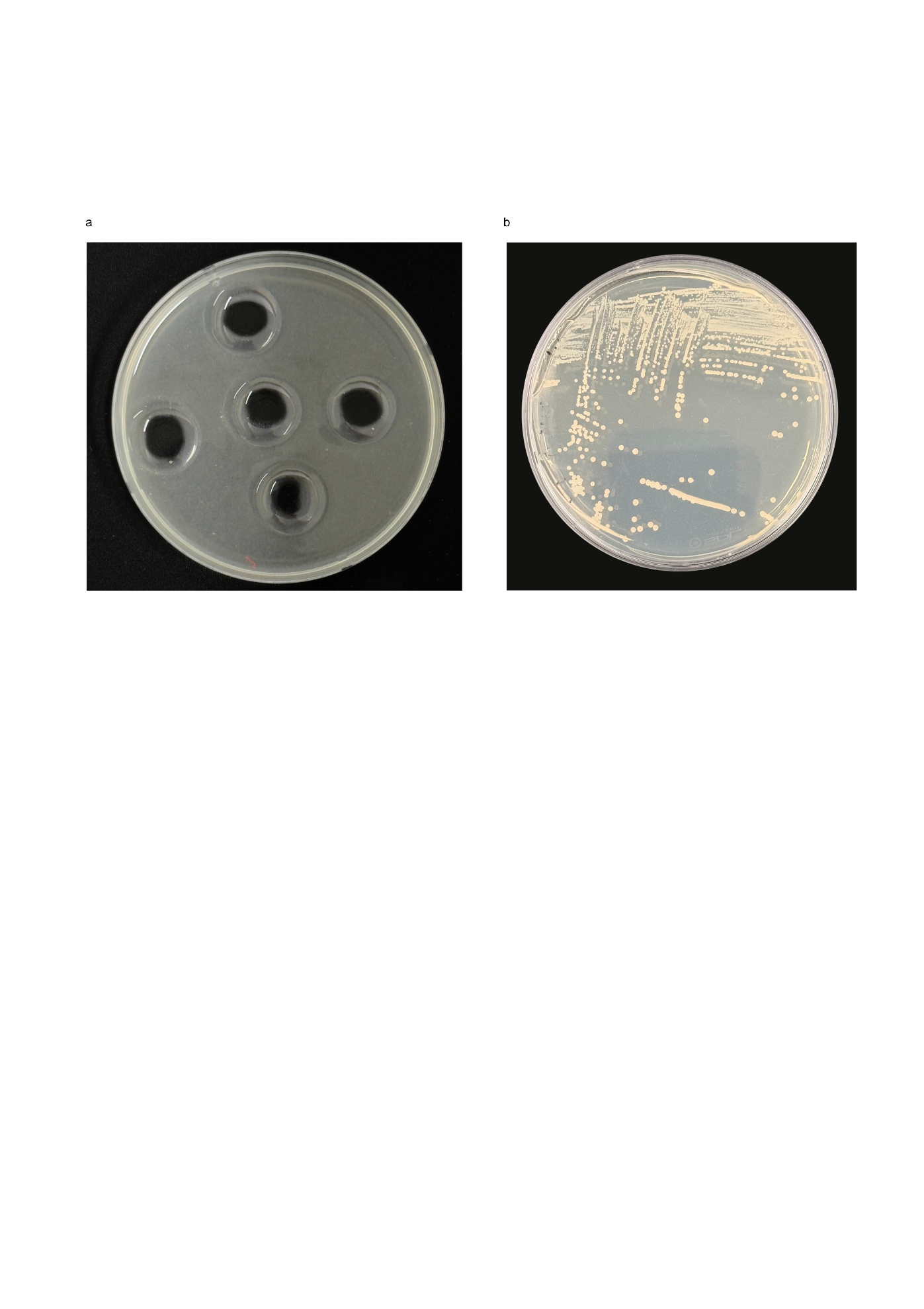


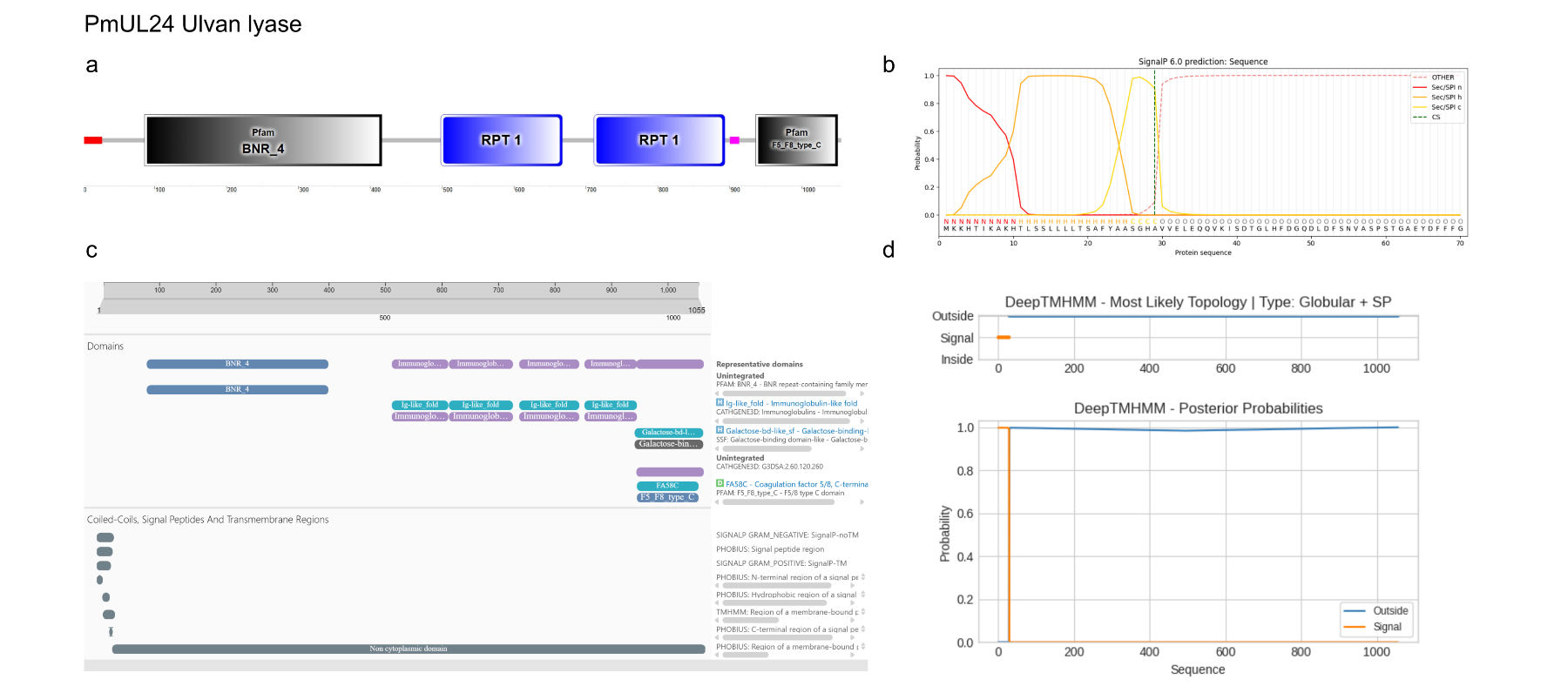


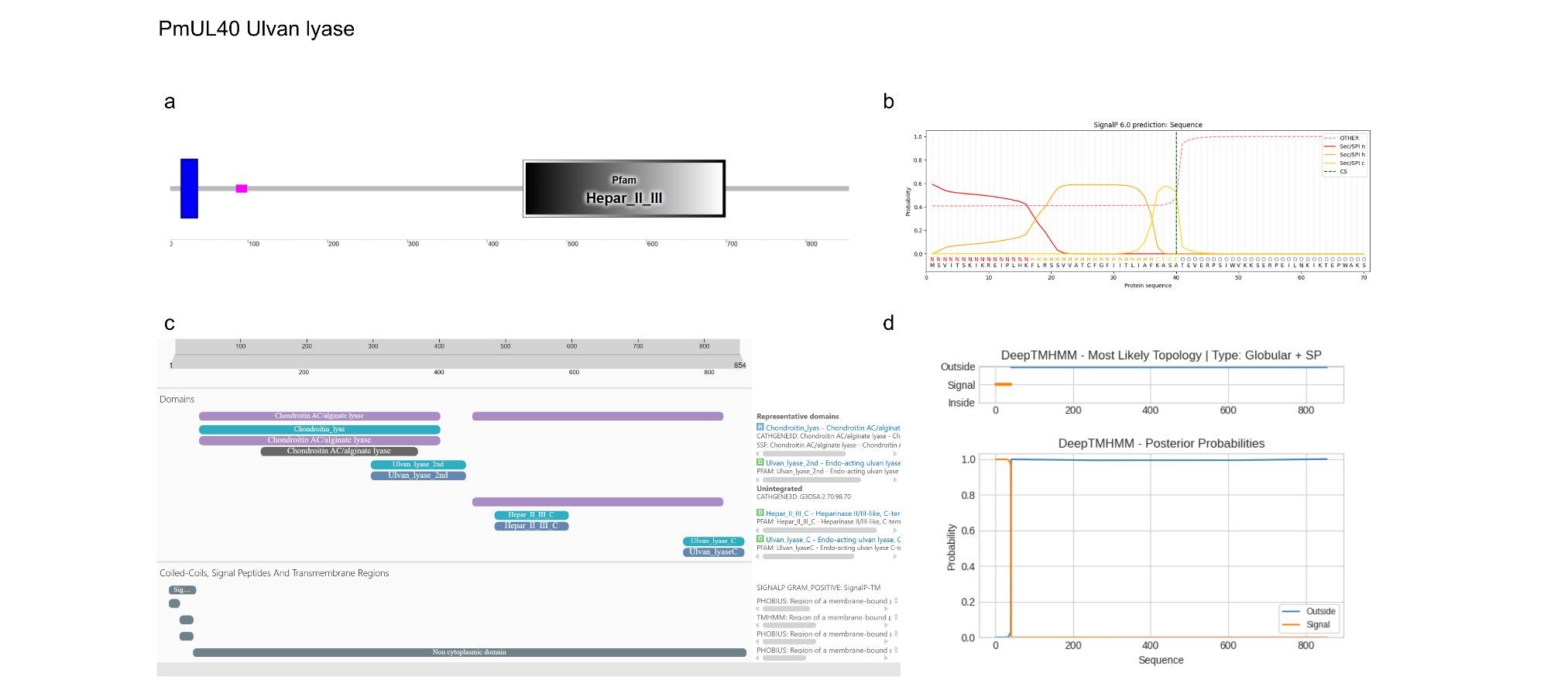

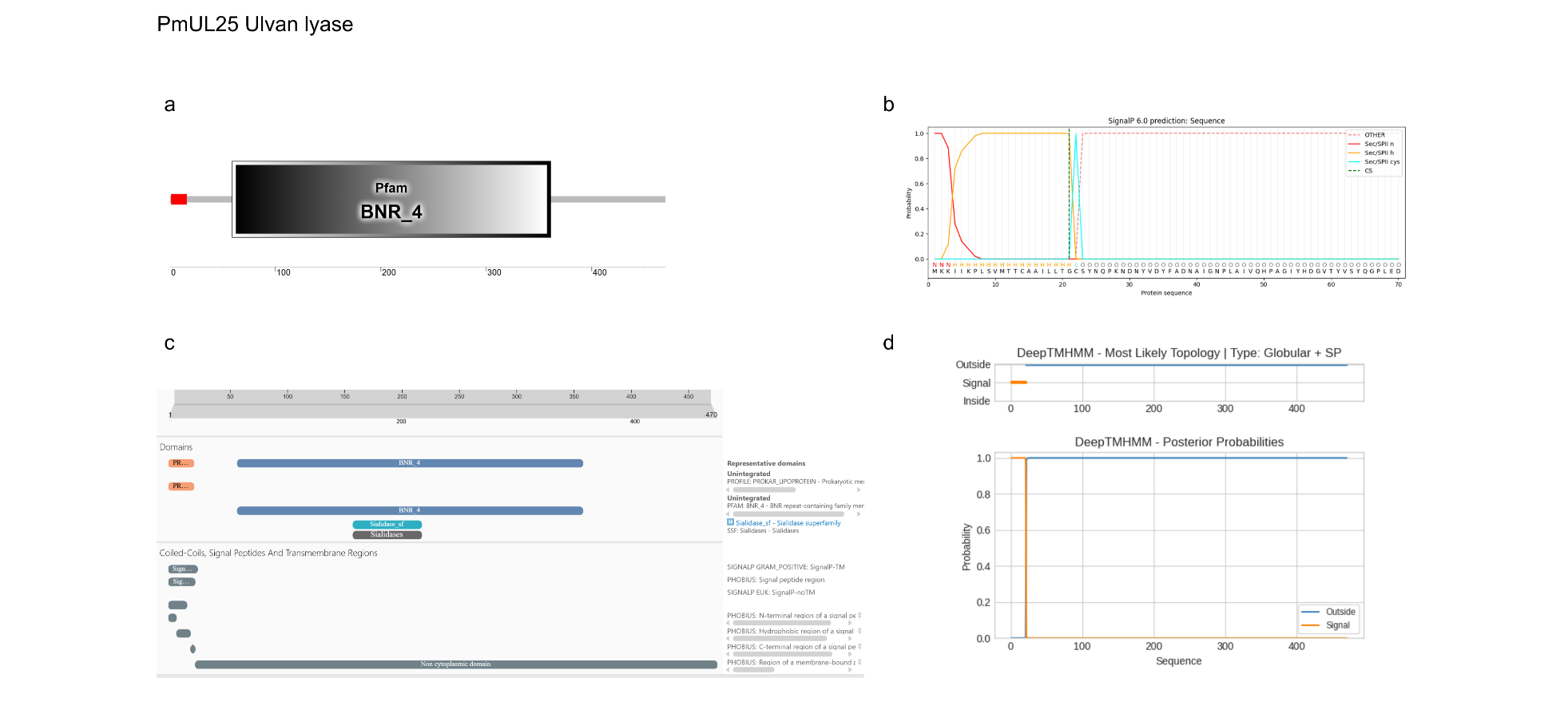


**Supplementary Figure 2: Computational analysis of ulvan lyase protein architecture and localization features.** Domain organization and subcellular localization predictions for PmUL24, PmUL25, and PmUL40 using multiple bioinformatics tools. **(a)** Functional domain architecture predicted by SMART (Simple Modular Architecture Research Tool), showing conserved catalytic domains and auxiliary regions. **(b)** Signal peptide prediction by SignalP 6.0, indicating the presence and cleavage sites of N-terminal secretion signals. **(c)** Protein domain and motif analysis by InterProScan, displaying comprehensive annotation of functional domains, family classifications, and conserved sequence features. **(d)** Transmembrane helix topology prediction by DeepTMHMM, illustrating membrane-spanning regions and protein orientation.


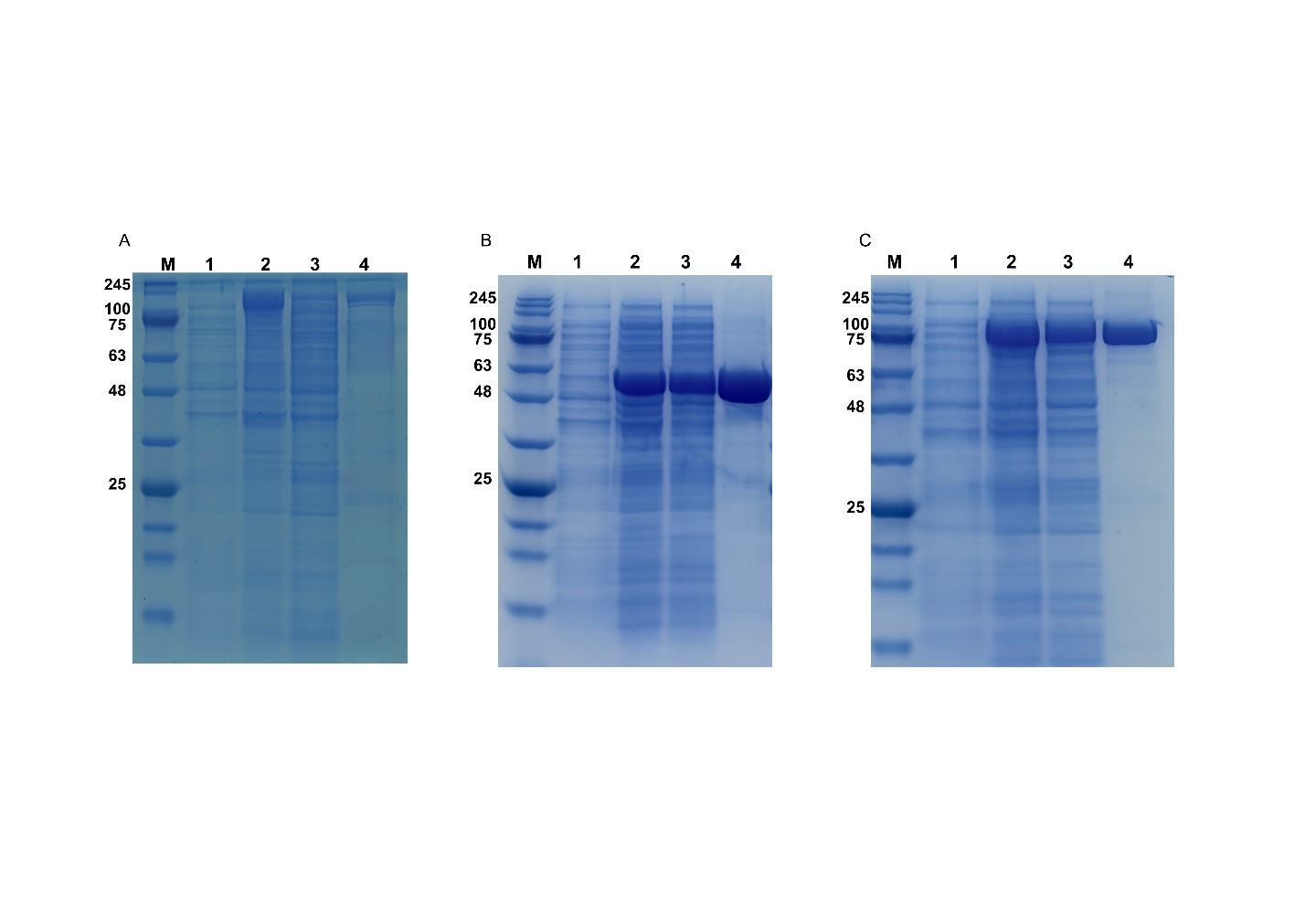


**Supplementary Figure 3:**  SDS-PAGE analysis of recombinant ulvan lyase expression and purification. Proteins were separated on 12% (w/v) SDS-PAGE gels and visualized by Coomassie Brilliant Blue staining. (A) PmUL24, (B) PmUL25, and (C) PmUL40. Lane M: prestained molecular mass marker; Lane 1: total cell lysate before isopropyl-β-D-thiogalactopyranoside (IPTG) induction; Lane 2: total cell lysate after IPTG induction; Lane 3: soluble fraction after IPTG induction; Lane 4: purified ulvan lyase. The prominent bands in Lane 4 correspond to the expected molecular masses of the recombinant enzymes, confirming successful expression and purification to apparent homogeneity.


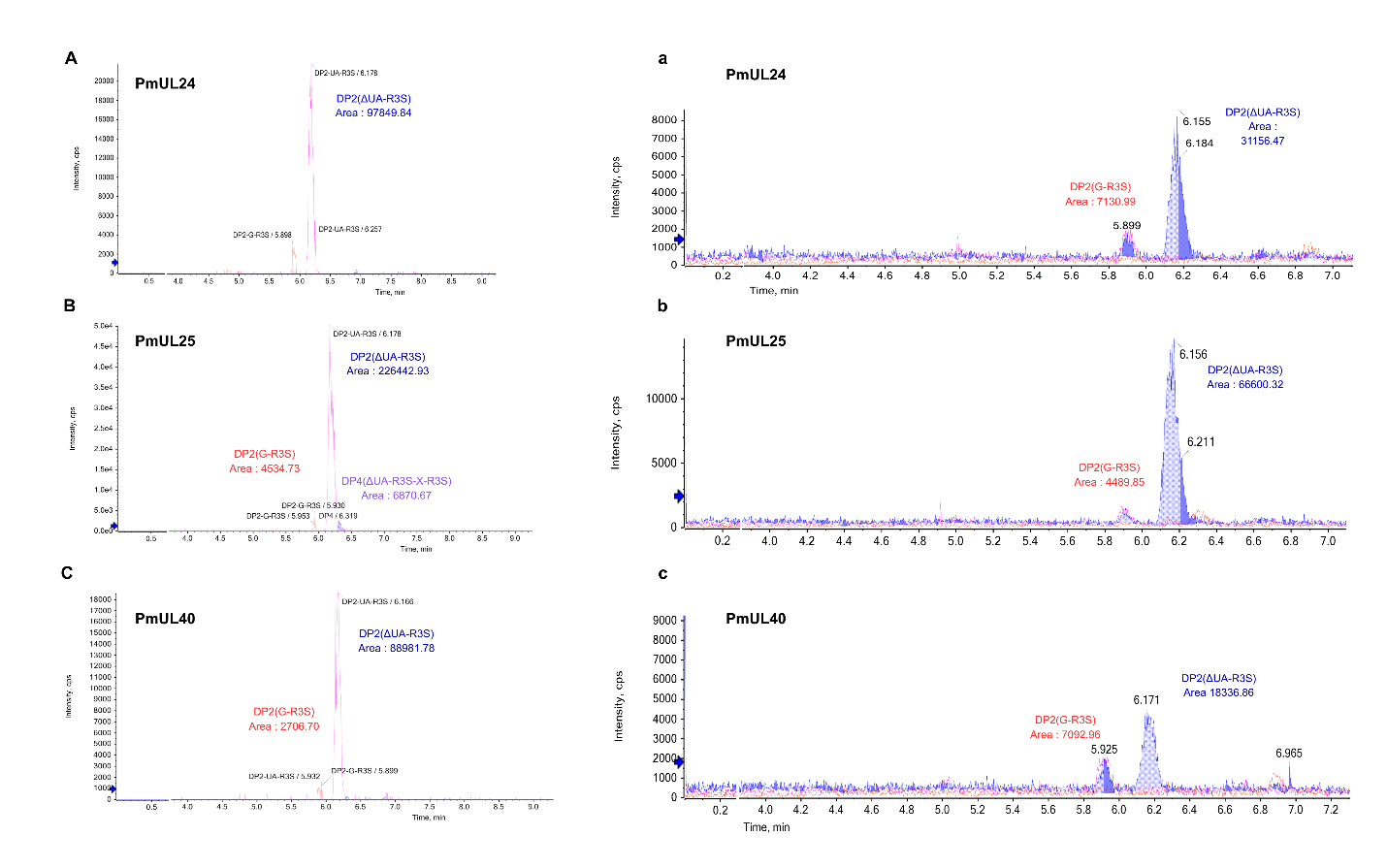


**Supplementary Figure 4: LC-MS analysis of ulvan depolymerization products generated by recombinant ulvan lyases.** Chromatographic profiles showing the oligosaccharide products from ulvan degradation analyzed by liquid chromatography-mass spectrometry. **(A, a)** PmUL24 products detected by Information-Dependent Acquisition (IDA) mode and Multiple Reaction Monitoring (MRM) mode, respectively. **(B, b)** PmUL25 products detected by IDA and MRM modes, respectively. **(C, c)** PmUL40 products detected by IDA and MRM modes, respectively. IDA mode provides comprehensive detection of all product ions, while MRM mode offers targeted quantification of specific disaccharide products DP2(ΔUA-R3S) and DP2(G-R3S). Major peaks correspond to unsaturated disaccharides, with PmUL25 uniquely generating tetrasaccharide DP4 oligomers in addition to the primary DP2 products. The complementary detection modes confirm the product specificity and enzymatic activity of each ulvan lyase.


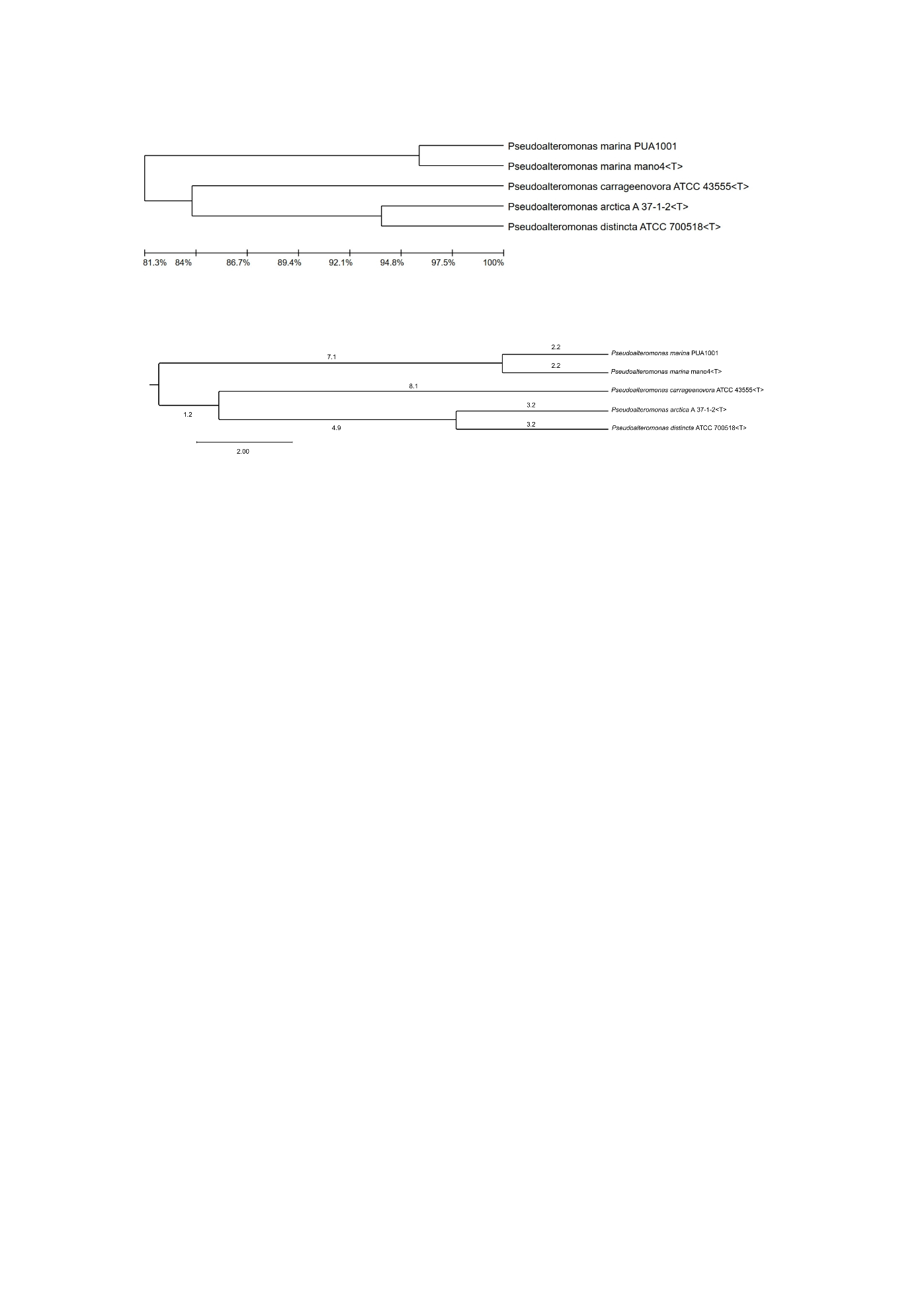


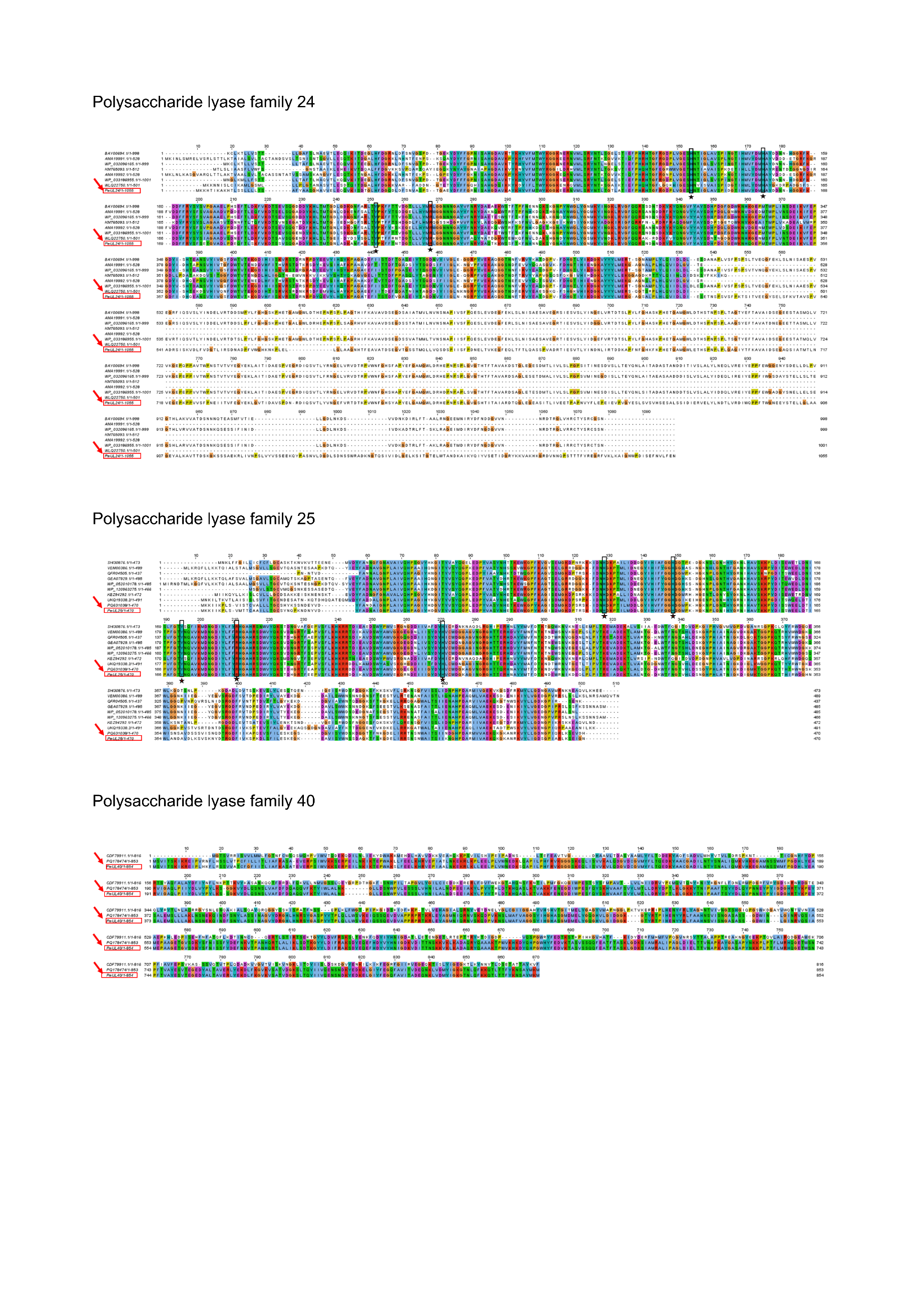


**Supplementary Figure 5: Multiple sequence alignment of characterized polysaccharide lyase families.** Multiple sequence alignments of ulvan lyase sequences from PL24, PL25, and PL40 families. All sequences represent biochemically characterized enzymes retrieved from the CAZy database and NCBI. Conserved catalytic residues are highlighted, and the degree of conservation is indicated by color intensity or consensus symbols. The alignments reveal family-specific conserved motifs and catalytically important residues essential for ulvan degradation.

**Supplementary Figure 6: OrthoANI-based phylogenetic analysis of** *Pseudoalteromonas marina* **strain PUA1001.** Phylogenetic tree constructed using OrthoANI (Average Nucleotide Identity) values to determine the taxonomic position and genomic relatedness of *P. marina* PUA1001 with other *Pseudoalteromonas* species and related genera. OrthoANI values are displayed at nodes, with values ≥95% indicating species-level identity. The analysis confirms the taxonomic classification of strain PUA1001 within the *Pseudoalteromonas* genus and reveals its closest genomic relatives. Branch lengths represent the degree of genomic divergence, and the tree topology reflects the evolutionary relationships among marine bacterial species.


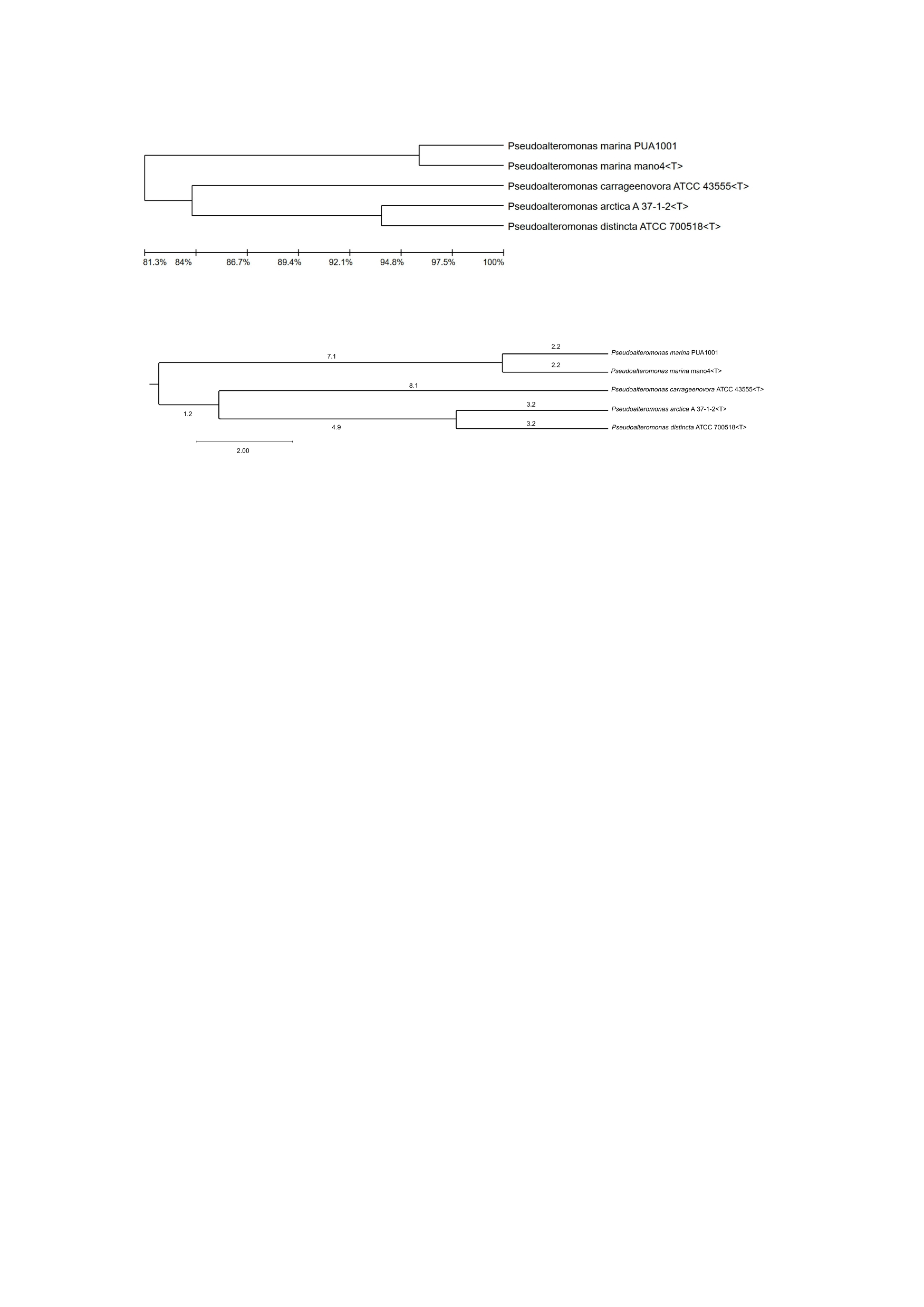


Supplementary Figure 7: **Pan genome analysis of** *Pseudoalteromonas* **species reveals core and accessory genome components.** Venn diagram illustrating the distribution of orthologous gene clusters across five *Pseudoalteromonas* genomes, including *P. marina* PUA1001. The diagram delineates the pan genome structure, showing the core genome (genes shared by all strains), accessory genome (genes present in two or more strains), and strain-specific genes (unique to individual genomes). Numbers indicate the quantity of gene clusters in each category. This comparative genomic analysis reveals the genetic diversity within the *Pseudoalteromonas* genus and highlights strain specific adaptations, including the ulvan utilization gene cluster unique to or enriched in *P. marina* PUA1001, reflecting niche specific metabolic capabilities related to algal polysaccharide degradation.


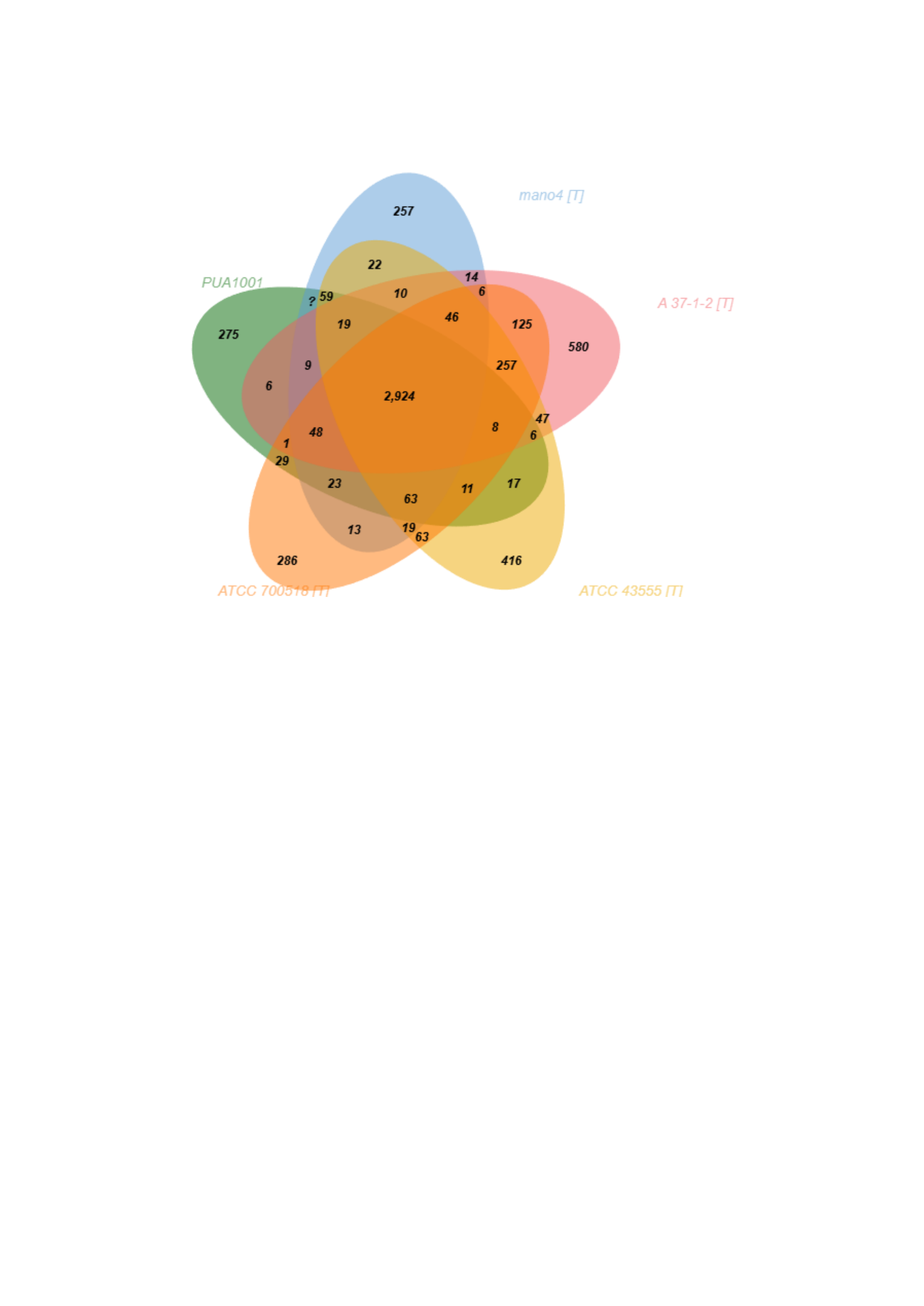

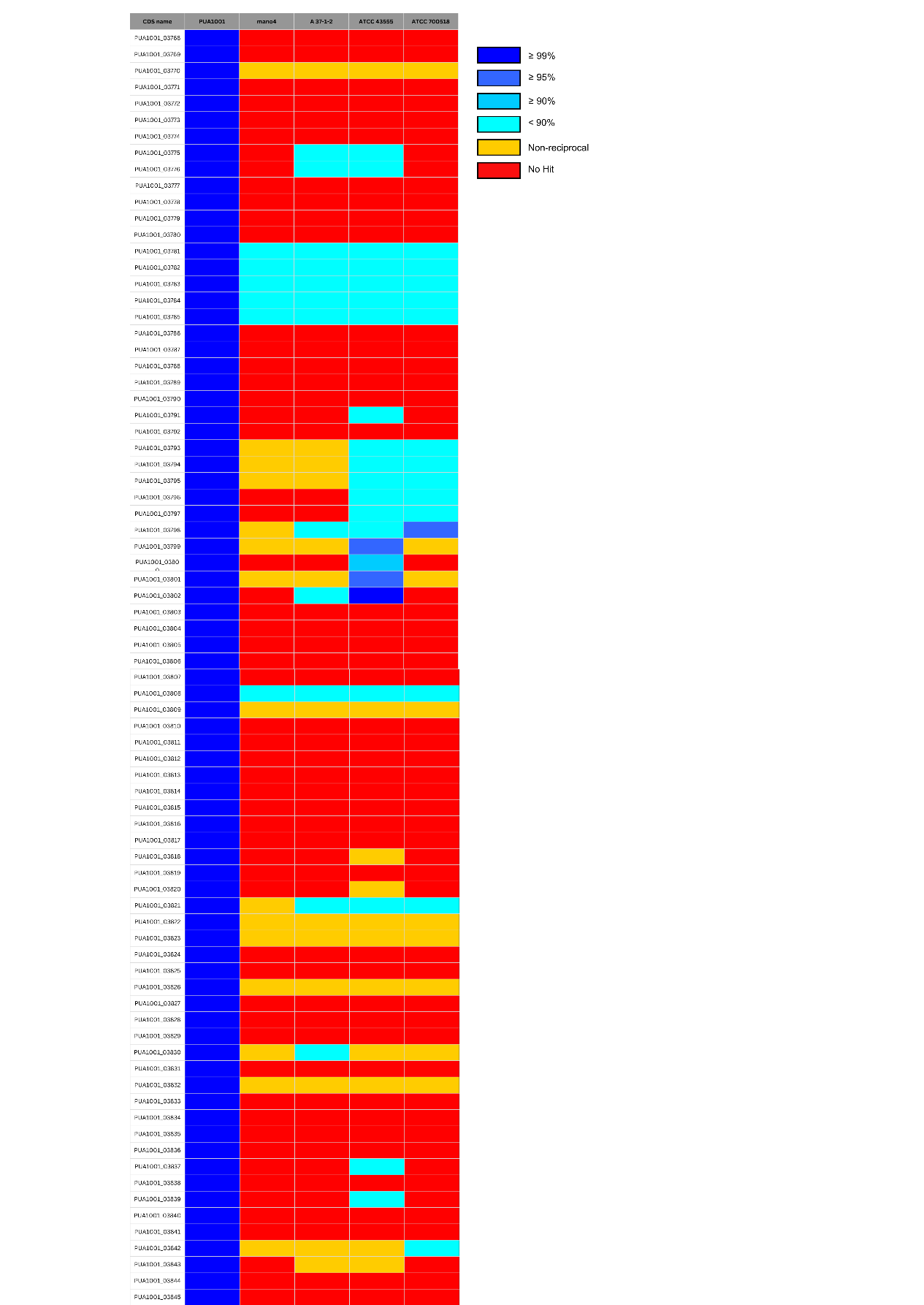


**Supplementary Figure 8: Pairwise ortholog matrix analysis of contig 3 genes across** *Pseudoalteromonas* **genomes.** Heatmap displaying the presence and absence of orthologous genes from contig 3 across the five *Pseudoalteromonas* genomes analyzed. Each row represents a gene from the *P. marina* PUA1001 contig 3, and each column represents a different *Pseudoalteromonas* strain. Color intensity or shading indicates the degree of sequence similarity or presence/absence of orthologs.
